# N-Acetylcysteine, Tiron, and Their Combination: In Vitro Antioxidant and Anti-Inflammatory Activities and Their Protective Effect in a Rat Model of Acetic Acid-Induced Ulcerative Colitis

**DOI:** 10.3390/ijms27167146

**Published:** 2026-08-10

**Authors:** Ahmed Kouki, Dorsaf Bouzazi, Asma Trabelsi, Lina Mbirki, Salwa Bouadballah, Safia El-Bok, Hamouda Beyrem, Maria Chiara Valerii, Enzo Spisni, Ezzedine Aouani, Mossadok Ben-Attia

**Affiliations:** 1 Environment Biomonitoring Laboratory (LR01/ES14), Sciences Faculty of Bizerte, University of Carthage, Zarzouna, Bizerte 7021, Tunisia; ahmed.kouki@fsb.ucar.tn (A.K.); asma.trabelsi@fsb.ucar.tn (A.T.); salwa.bouabdallah@fsb.ucar.tn (S.B.); hamouda.bayrem@fsb.ucar.tn (H.B.); mossadok.benattia@fsb.ucar.tn (M.B.-A.); 2 Cardiovascular Physiology and Pathophysiology Platform, Laboratory of Biomolecules, Venoms and Theranostic Applications, (LR20IPT01), Institut Pasteur of Tunis, Tunis El Manar University, Tunis 1068, Tunisia; dorsaf.bouzazi@pasteur.utm.tn; 3 Laboratory of Integrated Physiology (LR17ES02), Faculty of Sciences of Bizerte, University of Carthage, Bizerte 7021, Tunisia; linambirki@gmail.com; 4 Laboratory of Biodiversity, Biotechnologies and Climate Change (LR11/ES09), Faculty of Sciences of Tunis, University of Tunis El-Manar, Tunis 2092, Tunisia; safia.elbok@fst.utm.tn; 5 Department of Biological, Geological and Environmental Sciences, University of Bologna, Via Selmi 3, 40126 Bologna, Italy; mariachiara.valerii2@unibo.it; 6 Laboratory of Bioactive Substances (LR03BBC15), Center of Biotechnology of Borj-Cedria, BP 901, Hammam-Lif 2050, Tunisia; ezzedine_aouani@yahoo.fr

**Keywords:** N-acetylcysteine, Tiron, ulcerative colitis, oxidative stress, inflammatory response

## Abstract

Ulcerative colitis is characterized by inflammation, oxidative stress, and excessive free radical production. This study investigated the antioxidant, anti-inflammatory, and protective effects of N-acetylcysteine (NAC), Tiron, and their fixed-ratio combination in acetic acid-induced colitis. An integrated approach was used, combining ligand–ligand docking, acellular antioxidant and protein-denaturation assays, and an in vivo model in male Wistar rats. Colitis was induced by intrarectal administration of 3% acetic acid after 14 days of intraperitoneal pretreatment with NAC, Tiron, or NAC–Tiron. Docking analysis suggested physicochemical compatibility through non-covalent interactions. In vitro, NAC, Tiron, and their combination showed antioxidant and anti-denaturation activities. NAC–Tiron displayed greater activity than the individual compounds in selected endpoints, particularly 2,2-diphenyl-1-picrylhydrazyl (DPPH) radical scavenging, but did not consistently outperform them across the other assays. In vivo, NAC, Tiron, and NAC–Tiron attenuated macroscopic and histological colonic damage, reduced inflammatory cell infiltration and edema, decreased lipid peroxidation and protein carbonylation, and helped preserve superoxide dismutase (SOD) activity, reduced glutathione (GSH), and total thiols. The treatments also attenuated increases in C-reactive protein (CRP) and free iron without evident worsening of the measured systemic biochemical parameters. Overall, these findings provide an exploratory proof of concept for fixed-ratio NAC–Tiron co-administration but do not establish pharmacological synergy or superiority over standard therapies.

## 1. Introduction

Ulcerative colitis (UC), together with Crohn’s disease (CD), belongs to the spectrum of inflammatory bowel diseases (IBD), chronic relapsing–remitting disorders of the gastrointestinal tract characterized by alternating phases of active inflammation, clinical remission, and recurrent flare-ups [1,2,3]. Although currently available therapies, including corticosteroids, aminosalicylates, immunosuppressants, and biological agents such as anti-TNF-α antibodies, can be effective in inducing and maintaining remission, their long-term use may be limited by high costs, loss of response or treatment resistance, corticosteroid dependence, and adverse effects affecting patient adherence and therapeutic continuity [4,5,6]. Therefore, complementary or add-on strategies able to support mucosal protection, reduce oxidative and inflammatory burden, and lower the risk of relapse are of considerable interest.

The inflammatory process in UC is sustained by a complex interplay among epithelial barrier dysfunction, excessive production of reactive oxygen species, lipid and protein oxidation, and persistent activation of innate and adaptive immune responses [7,8,9,10,11].

Even during clinical remission, residual endoscopic or histological mucosal inflammation may persist and has been associated with a higher risk of relapse, suggesting that ongoing immune activation contributes to tissue vulnerability and disease reactivation [12,13]. These mechanisms are also reproduced, at least in part, in experimental models of chemically induced colitis, including acetic acid-induced colitis in rats, where mucosal injury is associated with granulocyte infiltration and increased production of hydrogen peroxide (H_2_O_2_), superoxide anion (O_2_^•−^), and other reactive species generated by mitochondrial respiration, xanthine oxidase, nitric oxide synthases, cyclooxygenases, and NADPH oxidases [14,15,16]. Moreover, intestinal epithelial cells contribute actively to the inflammatory cascade through the activation of nuclear factor-kappa B (NF-κB), which promotes the transcription of pro-inflammatory cytokines and pro-oxidant mediators [7,17,18]. In parallel, the impairment of endogenous antioxidant defenses, including enzymatic antioxidant systems and glutathione-dependent pathways, further amplifies redox imbalance. This condition favors oxidative damage to proteins and membrane lipids, contributing to protein carbonylation and lipid peroxidation, as reflected by increased malondialdehyde (MDA) levels [9,10,19]. Therefore, oxidative stress and inflammatory signaling represent interconnected targets for controlling mucosal injury and mitigating chronic intestinal inflammation. In this context, natural antioxidants and anti-inflammatory compounds could be considered as supportive preventive strategies aimed at reinforcing redox homeostasis, limiting immune overactivation, and protecting the intestinal mucosa from recurrent inflammatory injury. To date, numerous natural compounds have shown promising protective effects in animal models of intestinal inflammation by attenuating gastrointestinal inflammatory responses, modulating oxidative stress and inflammation-related signaling pathways, and improving both histological damage and clinical parameters of disease severity [9,20,21]. However, despite their biological potential, the translation of natural compounds into standardized therapeutic or preventive strategies remains challenging because of variability in composition, bioavailability, and dose standardization. These factors may compromise data reproducibility and make large-scale production difficult [22]. Synthetic molecules targeting similar oxidative and inflammatory pathways may offer a more standardized and reproducible approach. Compared with complex natural extracts, these compounds may provide greater chemical stability, dose control, and scalability, facilitating their development as supportive strategies against intestinal inflammation.

N-acetylcysteine and Tiron, chemically known as disodium 4,5-dihydroxybenzene-1,3-disulfonate, are of particular interest because they are well-characterized synthetic molecules with antioxidant and anti-inflammatory properties, acting through partially distinct and potentially complementary redox-related mechanisms. Previous studies have already investigated the biological effects of NAC and Tiron, showing that both molecules may act as promising antioxidant and anti-inflammatory candidates in different experimental settings [23]. NAC is widely recognized as a thiol-containing antioxidant and glutathione precursor, thereby contributing to the maintenance of intracellular redox homeostasis [24,25,26]. Beyond its direct redox activity, NAC has been reported to modulate Nrf-2- and MAPK-related pathways and to attenuate inflammatory responses by affecting matrix metalloproteinase activity, MAPK phosphorylation, and intercellular adhesion molecule-1 regulation [27,28]. Its protective effects have been documented in several in vivo models, including pulmonary disease, fibrosis, and intestinal inflammation [29,30,31,32,33]. Tiron, a highly water-soluble catechol derivative bearing sulfonic acid groups, is known for its metal-chelating and free-radical-scavenging properties [34,35,36]. By limiting mitochondrial reactive oxygen species, superoxide anion, peroxynitrite, and hydrogen peroxide, Tiron may protect cells from oxidative injury and modulate inflammatory pathways, including NF-κB, Nrf-2, NADPH oxidase-related signaling, and TNF-α release [37,38,39,40,41]. Combination strategies involving redox-active compounds are not unprecedented in experimental colitis or other inflammatory diseases. In 2,4,6-trinitrobenzenesulfonic acid (TNBS)-induced colitis, NAC has previously been administered together with mesalamine, and mesalamine has also been combined with antioxidants such as vitamin C and vitamin E [42,43]. Outside intestinal inflammation, NAC alone or in combination with vitamin E has been evaluated as an adjunct to standard psoriasis treatment, although the addition of vitamin E did not provide a statistically significant benefit over NAC alone [44]. Tiron has also been co-administered with glutathione in an experimental model of metal-induced oxidative stress, with effects that varied according to the endpoint examined [45]. These studies indicate that the general concept of combining antioxidant or redox-active agents is not novel and that co-administration does not necessarily result in a uniform additional benefit. However, to our knowledge, the specific NAC–Tiron combination has not previously been evaluated in experimental colitis. The rationale for testing this combination derives from the distinct chemical properties of the two compounds: NAC is a thiol-containing antioxidant and glutathione precursor, whereas Tiron is a water-soluble catechol derivative with radical-scavenging and metal-chelating properties. The present study should therefore be regarded as an exploratory assessment of this specific fixed-ratio combination rather than as the first antioxidant-combination strategy or as evidence of pharmacological synergy. In this study, we aimed to evaluate the antioxidant and anti-inflammatory properties of NAC, Tiron, and their combination (NAC–Tiron) using an integrated approach based on in silico molecular interaction analysis, in vitro antioxidant and protein-denaturation assays, and an in vivo model of acetic acid-induced colitis in rats. Specifically, we assessed the ability of these compounds to scavenge free radicals, chelate and reduce iron, limit protein denaturation, and protect against macroscopic and histological colonic damage, oxidative stress, inflammatory marker dysregulation, and extra-intestinal biochemical alterations. In particular, this approach was designed to characterize the antioxidant, anti-inflammatory, and protective effects of the fixed-ratio NAC–Tiron combination relative to the individual compounds, without assuming pharmacological synergy.

## 2. Results

### 2.1. In Silico Molecular Analysis of NAC–Tiron Interactions

**Figure 1:**
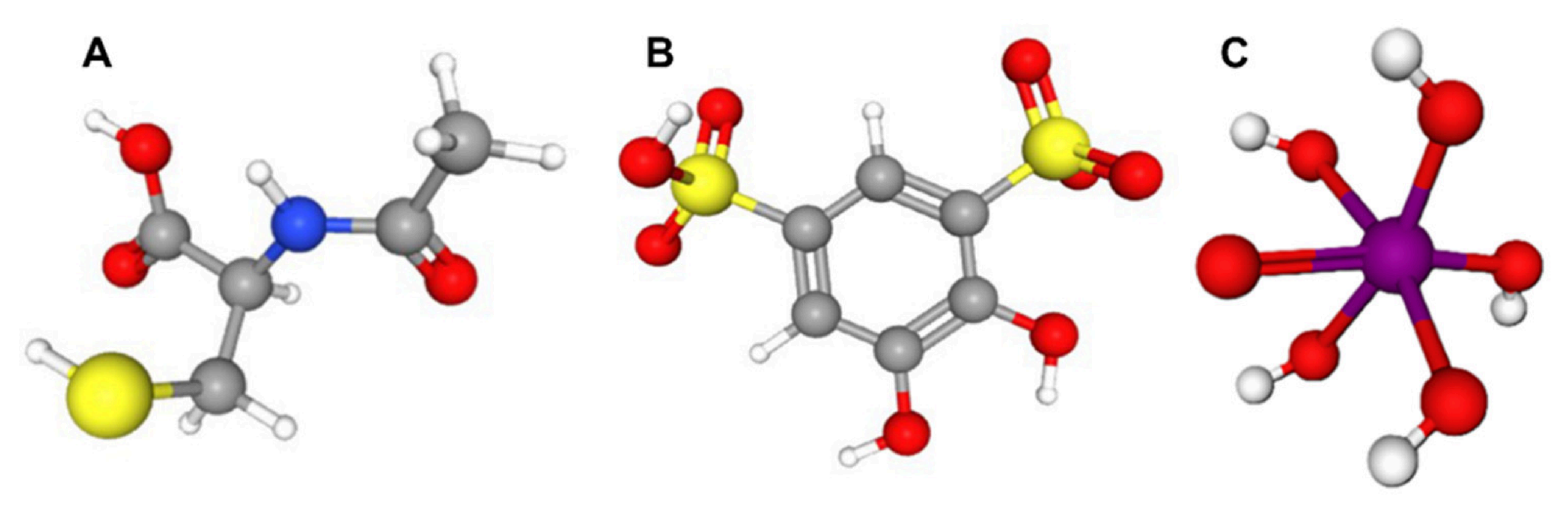
Optimized three-dimensional structures of the studied molecules. (**A**) NAC showing a flexible conformation with exposed thiol, amide, and carboxyl groups; (**B**) Tiron displaying a rigid aromatic scaffold with multiple sulfonate and hydroxyl groups; and (**C**) Periodic acid (H_5_IO_6_) characterized by a compact structure dominated by hydroxyl groups surrounding the iodine center. The atom color code is as follows: carbon: grey, hydrogen: white, oxygen: red, nitrogen: blue, sulphur: yellow and iodine: purple.

The optimized 3D structures highlighted distinct physicochemical features among the tested molecules (Figure 1). NAC adopted a flexible conformation with exposed thiol, amide, and carboxyl groups, which may favor polar and hydrogen-bond interactions. In contrast, Tiron displayed a more rigid aromatic scaffold enriched in hydroxyl and sulfonate groups, resulting in a highly polar molecular surface consistent with its metal-chelating and redox-active profile. Periodic acid showed a compact structure dominated by hydroxyl groups surrounding the iodine center, supporting its strong hydrophilic and oxidizing character. These structural features provided the basis for the subsequent exploratory ligand–ligand docking analysis and are summarized in Table 1.

**Figure 2:**
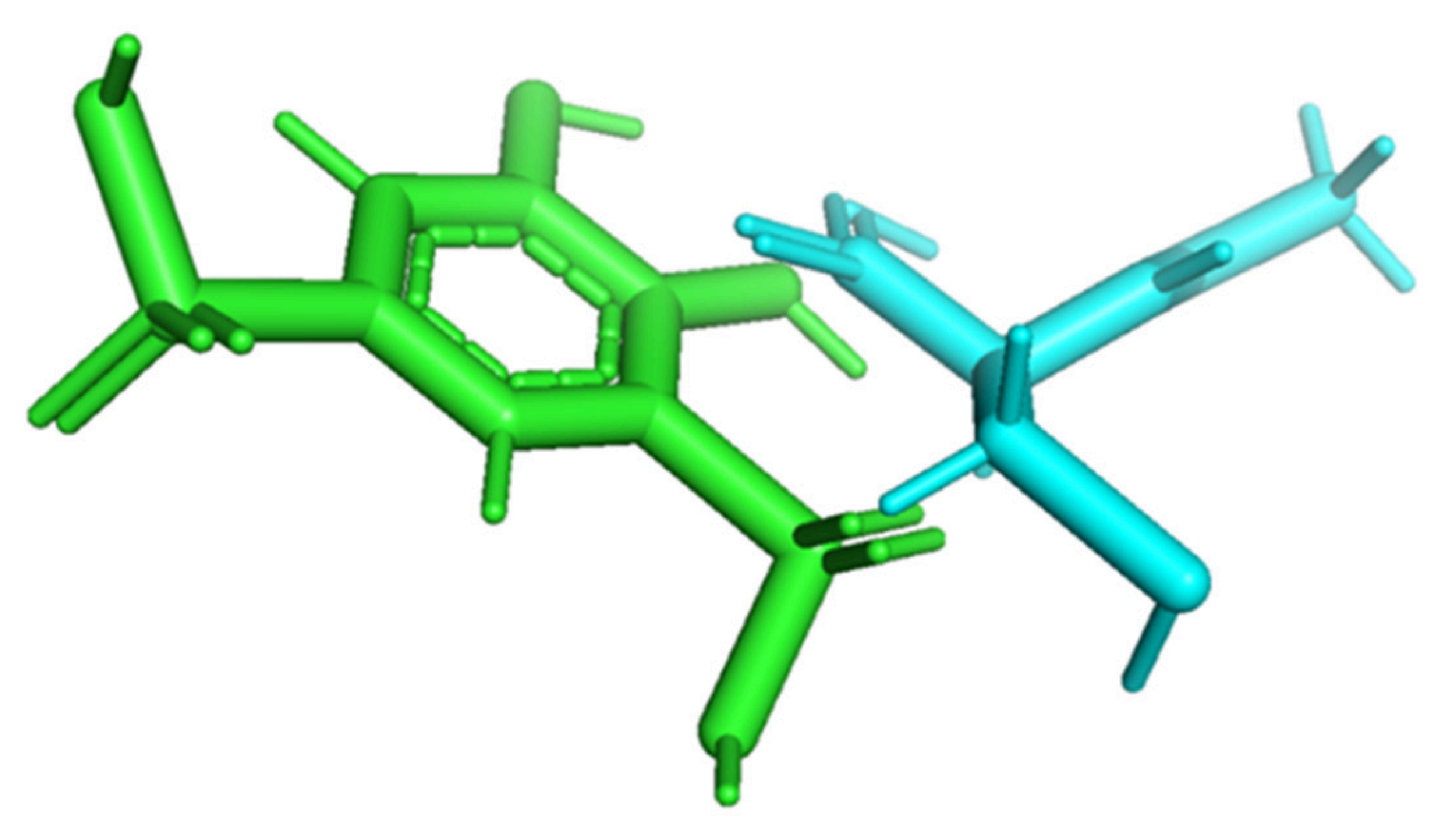
Structural complementarity and non-covalent contacts between NAC (light blue) and Tiron (green) obtained by molecular modelling, using PyMOL v3.0.

Docking simulations predicted that NAC may interact with Tiron through non-covalent contacts involving hydrogen bonds, electrostatic interactions, and van der Waals forces. These interactions mainly involved the hydroxyl and sulfonate groups of Tiron and the carboxylate, amide, and thiol-containing regions of NAC. Compared with the NAC–periodic acid complex, the NAC–Tiron complex showed a lower predicted binding energy and a more favorable ligand RMSD, suggesting a more defined interaction geometry under the computational conditions used (Table 2). Structural visualization further supported a close spatial arrangement between the polar functional groups of NAC and Tiron, with predicted non-covalent contacts distributed along complementary regions of the two molecules (Figure 2). Overall, these findings indicate physicochemical compatibility between NAC and Tiron and provided the rationale for evaluating the NAC–Tiron preparation in subsequent antioxidant, anti-inflammatory, and in vivo assays.

### 2.2. NAC, Tiron, and Their Combination Show Antioxidant and Anti-Denaturation Activities In Vitro

**Figure 3:**
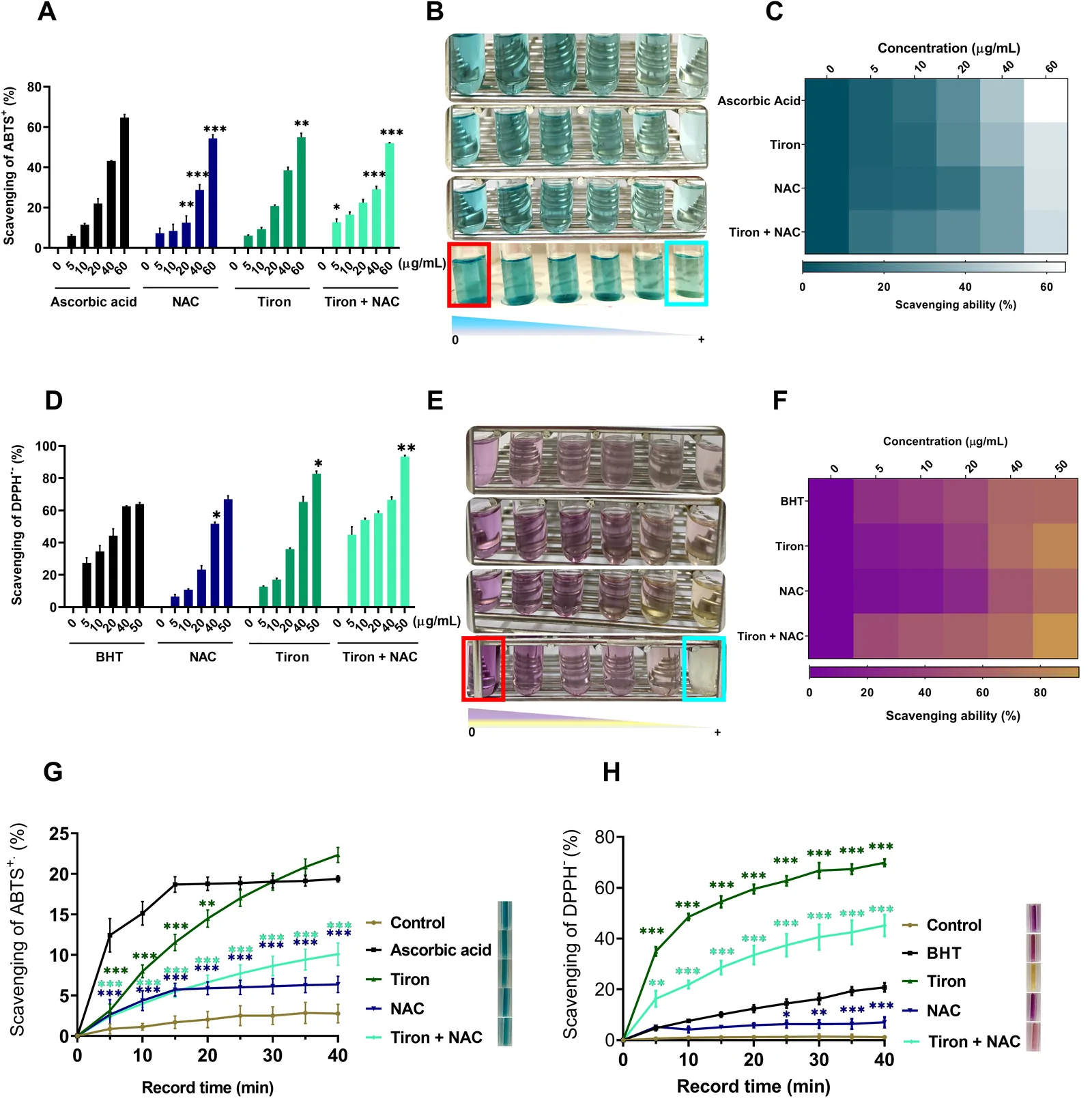
Effect of NAC, Tiron, and their combination (NAC–Tiron) on free-radical scavenging. All molecules were prepared at 1 mg/mL in PBS. The ABTS assay was performed to assess radical-scavenging activity (**A**), visually documented (**B**), and represented as a heat map (**C**). The DPPH assay was also performed (**D**), visually documented (**E**), and represented as a heat map (**F**). Kinetic analyses were used to assess the ability of NAC, Tiron, and their combination to scavenge ABTS^•+^ (**G**) and DPPH radicals (**H**) over 40 min. Results were obtained from three independent experiments and are expressed as mean ± S.E.M. * *p* < 0.05, ** *p* < 0.01, and *** *p* < 0.001 indicate comparisons between ascorbic acid and the tested preparations in the ABTS assay (**A**,**G**), and between BHT and the tested preparations in the DPPH assay (**D**,**H**).

The antioxidant potential of NAC, Tiron, and their combination (NAC–Tiron) using the 2,2′-azino-bis(3-ethylbenzothiazoline-6-sulfonic acid) (ABTS) test showed that, separately, these molecules exhibit ABTS^•+^ scavenging percentages comparable to the ascorbic acid reagent at low concentrations (5 and 10 µg/mL for NAC and from 5 to 40 µg/mL for Tiron) (Figure 3A). Moreover, increased concentrations of NAC and Tiron (40 and 60 µg/mL) triggered observable decolorization of the ABTS, confirming their ability to scavenge ABTS^•+^. Interestingly, the NAC–Tiron preparation (5 to 20 µg/mL) exhibited a potent ability to scavenge ABTS^•+^ compared to ascorbic acid. Moreover, we observed marked decolorization of the ABTS during the use of this combination at 60 µg/mL, confirming the antioxidant potential of this combination (Figure 3B,C).

In addition, the ability of these molecules to scavenge 2,2-diphenyl-1-picrylhydrazyl (DPPH^•−^) demonstrates that NAC, Tiron, and their combined form (NAC–Tiron) exhibit strong DPPH^•−^ scavenging activity, comparable to the BHT standard reagent, at prepared concentrations from 5 to 20 µg/mL (Figure 3D). This antioxidant capacity has been confirmed by the ability of these molecules, both individually and in combination, to decolorize the DPPH solution at concentrations of 40–60 µg/mL (Figure 3E,F). A kinetic method has been developed to assess the antioxidant capacities of NAC, Tiron, and their combination. Monitoring these molecules’ ability to scavenge ABTS^•+^ revealed that ascorbic acid reaches its peak capacity to neutralize ABTS^•+^ after 15 min of incubation (40 µg/mL, 20%). NAC preparation reached its maximum scavenging ability after 15 min (40 µg/mL, 7%), which was still significantly lower than ascorbic acid at the same concentration. However, Tiron exhibited a similar effect to ascorbic acid after 25 min. Importantly, NAC–Tiron (40 µg/mL) showed a notable ability to scavenge ABTS^•+^, slightly exceeding that of NAC alone (Figure 3G). The DPPH test showed that Tiron exhibited significantly greater DPPH radical-scavenging activity than BHT (*p* < 0.001). Moreover, NAC exhibited antioxidant activity that was still lower than that of the BHT standard reagent. Interestingly, the NAC–Tiron combination showed a potent ability to scavenge DPPH radicals, with an activity significantly higher than that of the BHT standard reagent. These findings indicate greater DPPH radical-scavenging activity of the NAC–Tiron preparation under the conditions of this assay. Because this effect was not consistently observed across the other antioxidant assays, it should not be interpreted as evidence of generalized superiority or pharmacological synergy (Figure 3H).

**Figure 4:**
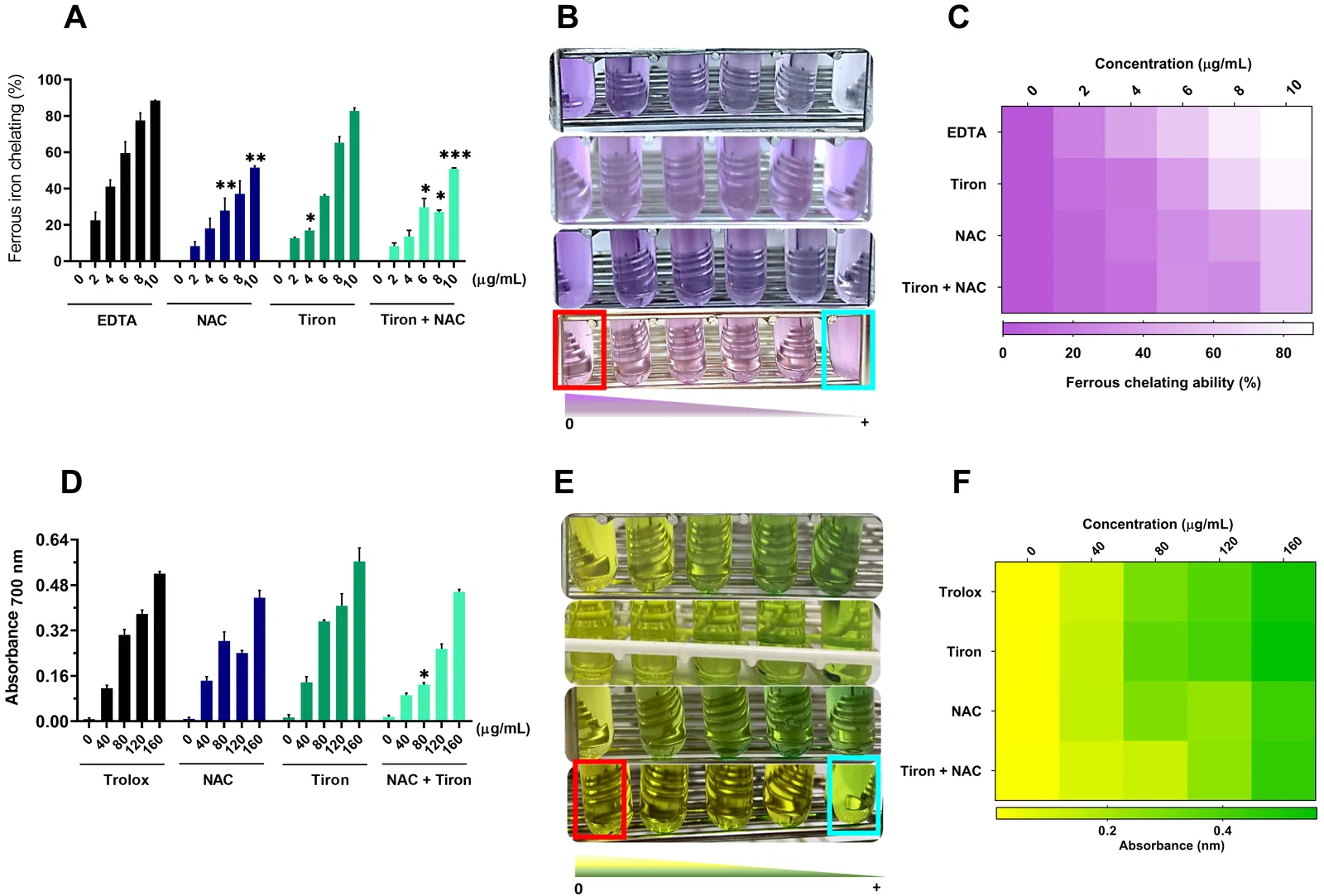
Control of the ferrous chelating ability and the ferric reducing antioxidant power of NAC, Tiron, and their combination (NAC–Tiron). All analyses are presented as mean ± S.E.M. (**A**,**D**), micrographs (**B**,**E**), and heat maps (**C**,**F**). Multiple approaches were used for each in vitro experiment to compare results with the standard reagents, with * *p* < 0.05, ** *p* < 0.01, and *** *p* < 0.001 (standard reagents vs. prepared molecules).

The ferrous iron chelation assay showed that Tiron, at 10 µg/mL, had a marked ability to chelate Fe^2+^ (82.782 ± 1.686%), reaching values comparable to those observed with ethylenediaminetetraacetic acid (EDTA) (88.403 ± 0.176%) (Figure 4A,B). The NAC–Tiron preparation at the highest concentration (10 µg/mL) exhibited a remarkable ability to chelate iron (50.929 ± 0.396%), comparable to that of the NAC molecule (Figure 4A,C). The ferric-reducing assay showed that NAC, Tiron, and NAC–Tiron displayed reducing activity, as indicated by their ability to reduce Fe^3+^ to Fe^2+^, compared with the reference antioxidant Trolox (Figure 4D). In line with this result, we observed an increase in absorbance measured at 700 nm (Figure 4E) and a color change from yellow to green, which correlated with the increase in concentration (Figure 4E,F).

**Figure 5:**
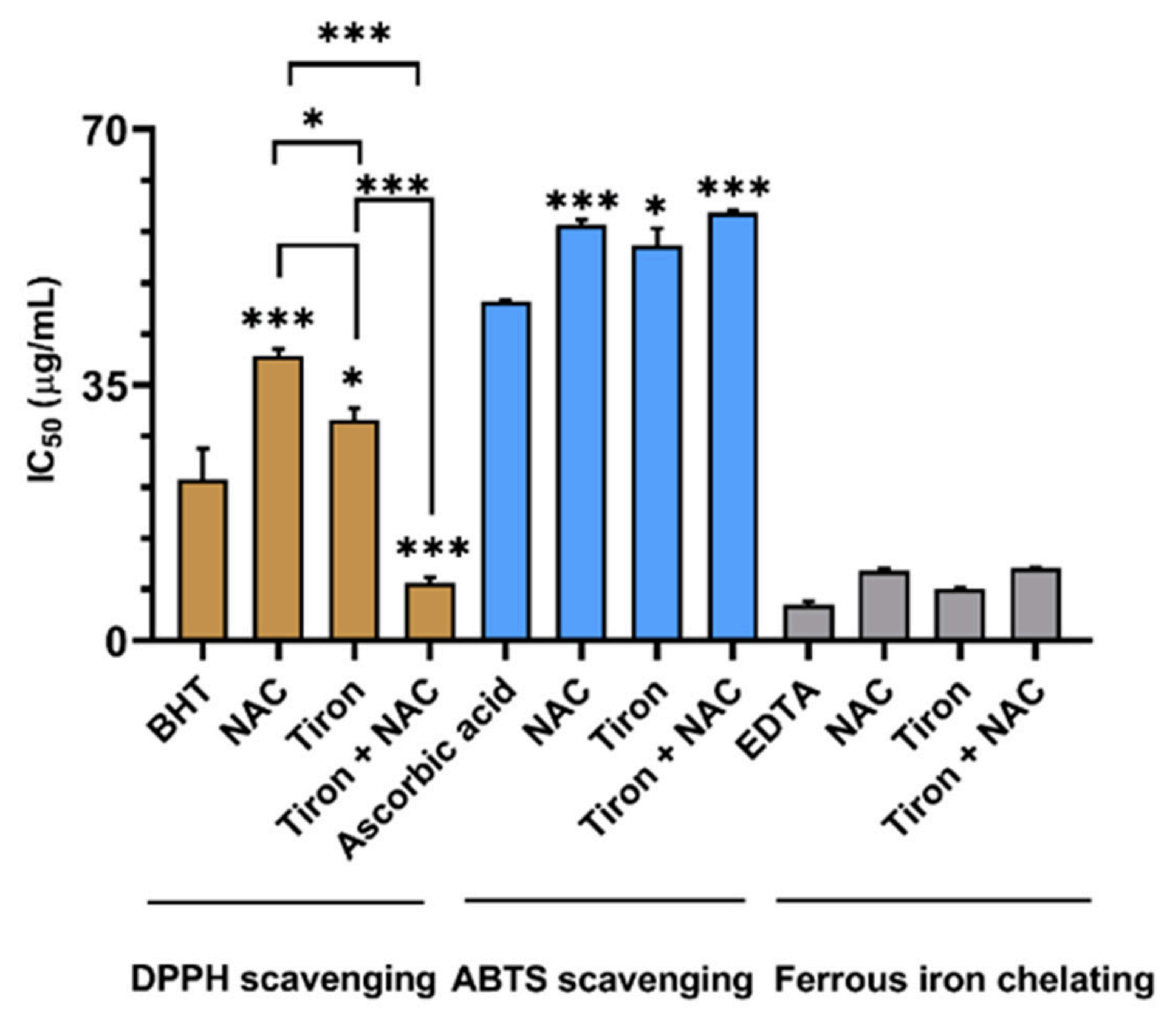
Antioxidant efficacies of those molecules (NAC, Tiron, and their association) using IC_50_ values for each antioxidant test. Data were presented as mean ± S.E.M. of three independent experiments, with * *p* < 0.05, and *** *p* < 0.001 indicating a comparison of standard reagents vs. prepared molecules.

IC_50_ values were measured by using the nonlinear regression method (Figure 5). The ABTS and DPPH tests. ABTS test showed that NAC exhibits an IC_50_ of 56.950 ± 0.648 µg/mL, Tiron shows an IC_50_ of 54.060 ± 2.384 µg/mL, and their combination results in an IC_50_ of 55.640 ± 0.250 µg/mL in scavenging ABTS^•+^. Moreover, the differences between these IC_50_ values were not statistically significant, supporting the antioxidant capacity of the NAC–Tiron combination in scavenging Cation radicals (Figure 5). DPPH test showed IC_50_ values of 38.985 ± 0.933 µg/mL for NAC and 30.213 ± 1.614 µg/mL for Tiron, significantly higher (*p* < 0.001) than those of BHT (IC_50_ = 22.076 ± 4.233 µg/mL). However, when combined (NAC–Tiron), IC_50_ was 7.924 ± 0.783 µg/mL, significantly lower than the values of NAC, Tiron, and BHT, indicating greater DPPH radical-scavenging activity of NAC–Tiron than NAC, Tiron, and BHT under the conditions of this assay (Figure 5). Moreover, IC_50_ values, showed no significant differences between those molecules and the EDTA reagent (Figure 4) nor between the NAC–Tiron combination (IC_50_ = 9.943 ± 0.024 µg/mL) and EDTA (IC_50_ = 4.906 ± 0.483 µg/mL) were observed. Overall, these findings support the ability of the NAC–Tiron combination to scavenge free radicals and chelate ferrous iron, in agreement with the results shown in Figure 3 and Figure 4.

**Figure 6:**
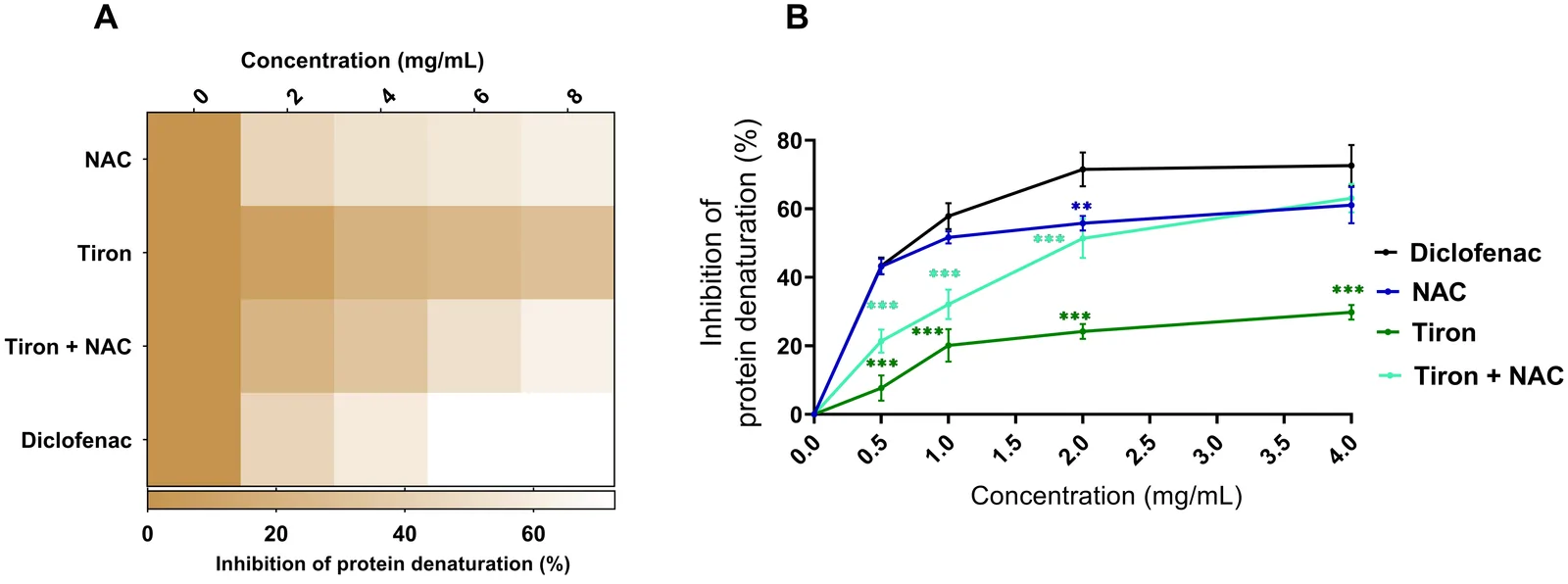
Assessment of the in vitro anti-inflammatory properties of NAC and Tiron and their association (NAC–Tiron) by controlling their ability to limit protein denaturation. Results were obtained in three independent experiments and presented as a heat map (**B**) and as mean ± S.E.M. (**A**) with ** *p* < 0.01 and *** *p* < 0.001 compared to the diclofenac reagent.

As shown in Figure 6, the protein-denaturation assay used to assess the preliminary anti-inflammatory potential of NAC, Tiron, and NAC–Tiron. NAC showed marked anti-denaturation activity at 4 mg/mL, with an IC_50_ value of 0.733 ± 0.070 mg/mL, comparable to that of diclofenac (0.698 ± 0.081 mg/mL). However, the effectiveness of Tiron in preventing protein denaturation was significantly lower (*p* < 0.001) than that of diclofenac (IC_50_ > 4 mg/mL). The NAC–Tiron combination retained anti-denaturation activity, with an IC50 of 2.114 ± 0.563 mg/mL. However, this value was numerically higher than those obtained for NAC and diclofenac, and the assay did not establish superior activity of the combination over NAC alone (see Figure 6A,B).

### 2.3. NAC, Tiron, and Their Combination Alleviate Induced Colitis in Rats

**Figure 7:**
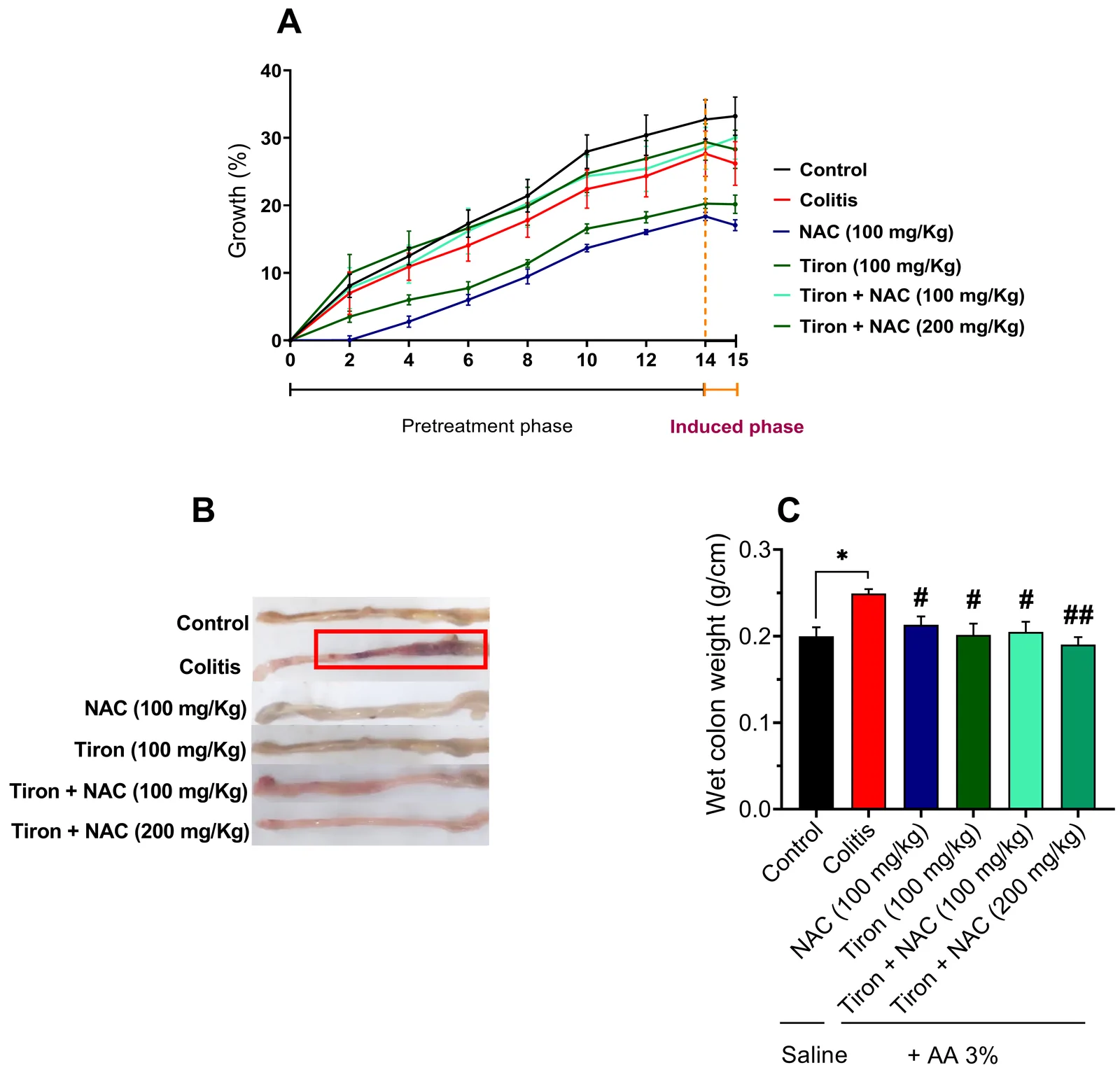
Effect of subacute Pretreatments with NAC, Tiron, and their combination on morpho-physiological signs in colitis induced by AA (3%). Rats were pretreated for 14 days with NAC (100 mg/kg, i.p.), Tiron (100 mg/kg, i.p.), or NAC–Tiron at total doses of 100 mg/kg (50 mg/kg NAC plus 50 mg/kg Tiron, i.p.) or 200 mg/kg (100 mg/kg NAC plus 100 mg/kg Tiron, i.p.) before the intrarectal administration of 3% AA. Rat growth (**A**), macroscopic damage of the colon (**B**) and the wet colonic weight (**C**) were monitored. Data are presented as mean ± S.E.M. of n = 6 rats per cage. * *p* < 0.05 indicates comparisons between control rats and colitis. # *p* < 0.05 and ## *p* < 0.01 indicate comparisons between the colitis group and other treated groups.

During the Pretreatment phase, all rats showed an increase in body weight. Control rats showed a notable increase in their body weight by 32.7 ± 2.9%, and the colitis group showed an augmentation by 27.7 ± 3.3%. In all animals, body weight growth remained within the range of 18–29% during the 14 days of treatment before colitis induction. After colitis induction, we noted a slight reduction in body weight in the animals with colitis. Only in the groups of rats pretreated with the combination (NAC–Tiron) at 100 and 200 mg/Kg the body weight was not found to be decreased after colitis (Figure 7A).

Macroscopic observation of the diseased group (colitis group) revealed marked inflammatory signs, including colon redness and thickening (blue square), accompanied by a significant increase in wet colon weight (from 0.227± 0.004 to 0.249 ± 0.007 g/cm, *p* < 0.05 vs. control rats). However, NAC (100 mg/kg, i.p.), Tiron (100 mg/kg, i.p.), and their combination at 100 mg/kg mitigated these pathological signs and decreased (*p* < 0.05) the wet colon weight vs. colitis group. Furthermore, the NAC–Tiron combination (200 mg/kg, i.p.) significantly reduced (*p* < 0.01) the wet colon weight (0.1901 ± 0.008 g/cm) compared to colitis rats (Figure 7B,C).

### 2.4. Treatment with NAC, Tiron, and Their Combination Protects Rats from Colitis Histopathological Damage

**Figure 8:**
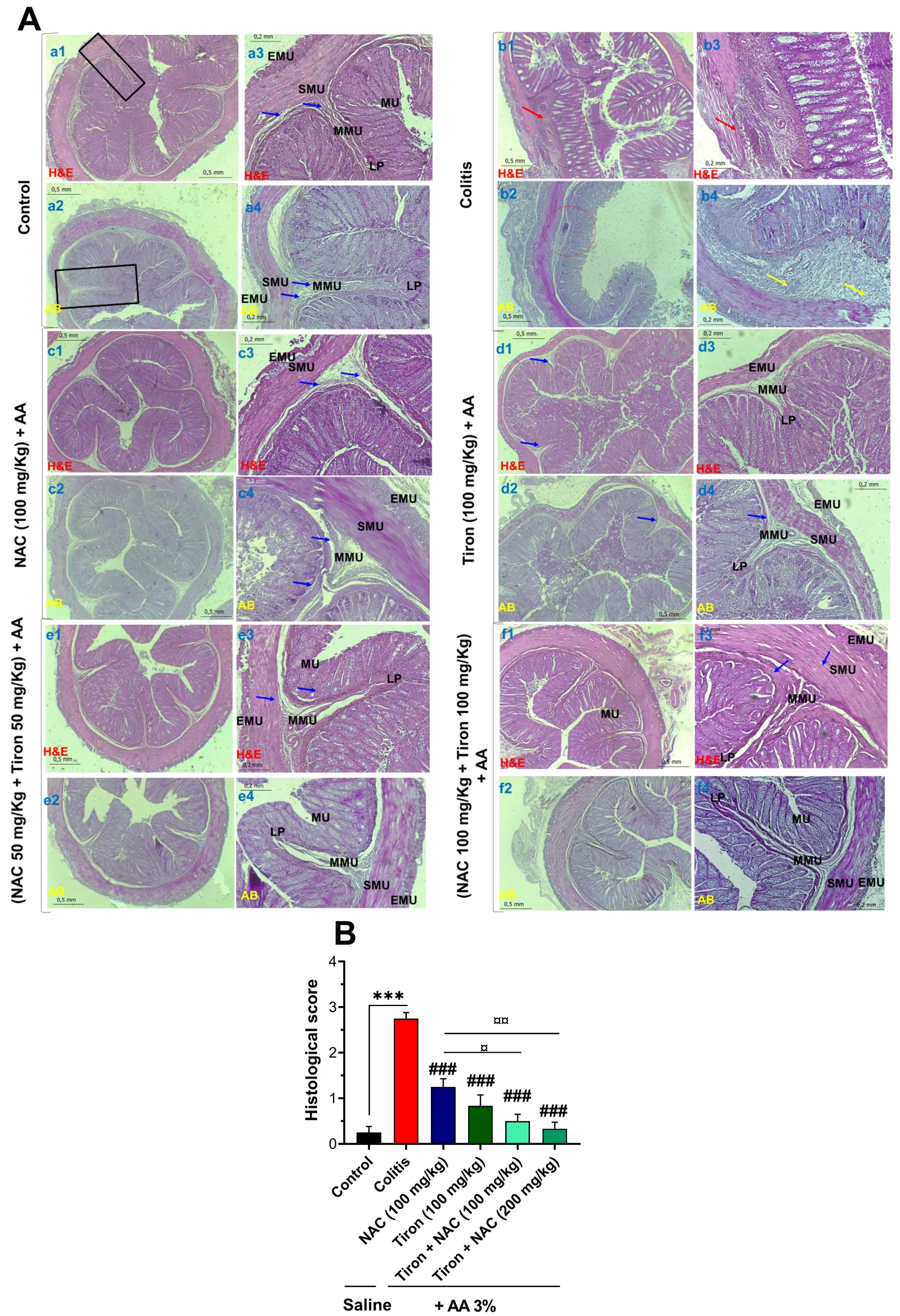
Assessment of NAC, Tiron, and their combination (NAC–Tiron) on histopathological damage in ulcerative colitis induced by AA 3% in rats. Rats received daily pretreatment for 14 days with NAC (100 mg/kg, i.p.), Tiron (100 mg/kg, i.p.), or NAC–Tiron at total doses of 100 mg/kg (50 mg/kg NAC plus 50 mg/kg Tiron, i.p.) or 200 mg/kg (100 mg/kg NAC plus 100 mg/kg Tiron, i.p.) before the intrarectal administration of 3% AA. Colonic tissues were harvested and microscopically examined using H.E. and A.B staining methods (**A**), and a histological score was then applied (**B**). Results were observed under two magnifications (×4 and ×10) with EMU: external mucosa, SMU: submucosal mucosa, MMU: muscularis mucosae, MU: mucosa, and LP: lamina propria. Blue arrow: normal epithelial structure, red arrow: altered epithelial structure, black square: normal villi, red circle: inflammatory cell accumulation, and yellow arrow: presence of edema. For the histological score, results were expressed as mean ± S.E.M. (n = 6), with *** *p* < 0.001 indicating comparisons between control and colitis. ### *p* < 0.001 indicates comparisons between the rat with colitis group and Pretreated rats. ¤ *p*< 0.05 and ¤¤ *p* < 0.01 indicate comparisons between Pretreated groups.

Histological investigation showed that, in the colitis group, the colon was marked by an altered epithelial structure (Figure 8A). In particular, we observed reduced villi length (Figure 8A(b1,b2)), inflammatory cell accumulation (red circle), and edema (yellow arrow), with non-distinctive epithelial zones (Figure 8A(b3,b4)). This was evident in comparison with normal colonic tissue (Figure 8A(a1,a2)), with healthy villi (black square); an intact microstructure, as evidenced by the presence of epithelial mucosa (EMU), submucosal mucosa (SMU), and muscularis mucosa (MMU); and an intact lamina propria (Figure 8A(a3,a4)). Colonic tissue of rats pretreated with NAC (100 mg/kg) (Figure 8A(c1–c4)) and Tiron (100 mg/kg) (Figure 8A(d1–d4)) showed a preserved colonic histological structure. This is shown by the presence of villi (blue arrow, Figure 8A(c1,c2), Figure 8A(d1,d2)), partial preservation of colonic epithelial tissue (presence of EMU, MU, LP, and MMU zones), and a reduction in inflammatory zones (Figure 8A(c3,c4,d3,d4)). In the combined treatment group, (NAC–Tiron) at 100 (Figure 8A(e1–e4)) and 200 mg/kg (Figure 8A(f1–f4)) colonic tissue showed a normal structure, marked by a well-organized epithelial zone (Figure 8A(e1,e2,f1,f2)), a reduced inflammatory cell infiltration, and normal villi in terms of structure and length (Figure 8A(e3,e4.f3.f4)). Histological scoring (Figure 8B) confirmed a marked increase in tissue damage in colitic rats compared with control animals (*p* < 0.001). Pretreatment with NAC, Tiron, or NAC–Tiron significantly reduced the histological score compared with the colitis group (*p* < 0.001). In addition, selected pairwise comparisons among the pretreated groups reached statistical significance, as indicated in Figure 8B.

### 2.5. NAC, Tiron, and Their Combination Modulate Oxidative Stress in Colitis

**Figure 9:**
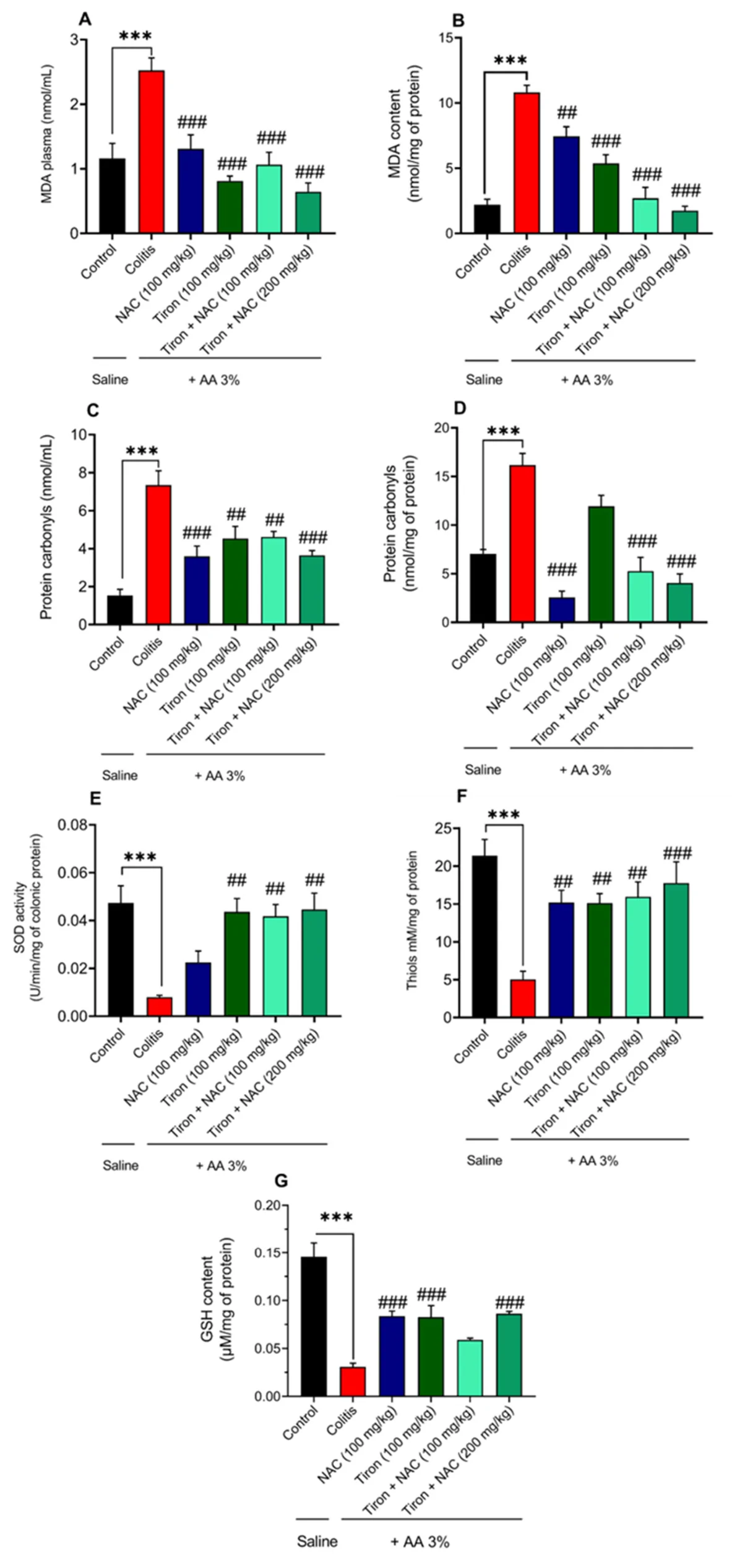
Effect of NAC, Tiron, and their association on plasma and tissue oxidant markers in colitis in rats induced by AA 3% administered intra-rectally. Animals received intraperitoneal pretreatment for 14 days with NAC (100 mg/kg), Tiron (100 mg/kg), or NAC–Tiron at total doses of 100 mg/kg (50 mg/kg NAC plus 50 mg/kg Tiron) or 200 mg/kg (100 mg/kg NAC plus 100 mg/kg Tiron) before colitis induction. Pro-oxidant markers, including lipid peroxidation in plasma (**A**) and colonic supernatants (**B**), were determined by measuring MDA content. The carbonylation of protein in plasma (**C**) and tissue (**D**) was determined. Antioxidant markers, including colonic SOD activity (**E**), total thiols (**F**), and GSH contents (**G**), were monitored. All results were expressed as mean ± S.E.M. of n = 6 rats per group. *** *p* < 0.001 compared to the control group, and ## *p*  < 0.01 and ### *p*  < 0.001 compared to the colitis group.

Plasma malondialdehyde (MDA) levels increased after colitis induction, rising from 1.162 ± 0.232 nmol/mL in control rats to 2.524 ± 0.195 nmol/mL in the colitis group (*p* < 0.001). In colonic tissue, MDA levels increased from 2.196 ± 0.429 nmol/mL in control rats to 10.811 ± 0.548 nmol/mL in colitic rats. Pretreatment with NAC (100 mg/kg) significantly reduced MDA levels in both plasma (1.311 ± 0.215 nmol/mL, *p* < 0.001) and colonic tissue supernatants (7.449 ± 0.731 nmol/mL) compared with the colitis group. Similarly, Tiron pretreatment significantly reduced MDA levels in both plasma and colonic tissue (*p* < 0.001 vs. colitis group). Finally, co-administration of NAC and Tiron at both tested doses (100 and 200 mg/kg) markedly reduced plasma and colonic MDA levels (*p* < 0.001 vs. colitis group) (Figure 9A,B). Protein carbonyl content also increased markedly after colitis induction. Plasma protein carbonyl levels rose from 1.539 ± 0.316 nmol/mL in control rats to 7.345 ± 0.756 nmol/mL in the colitis group (*p* < 0.001), whereas colonic tissue protein carbonyl levels increased from 7.044 ± 0.448 to 16.162 ± 1.215 nmol/mg protein (*p* < 0.001). Pretreatment with NAC, Tiron, or NAC–Tiron induced a marked and comparable reduction in plasma protein carbonyl levels compared with the colitis group (*p* < 0.001) (Figure 9C). In colonic tissue, protein carbonyl levels were significantly reduced after pretreatment with NAC or with both NAC–Tiron combinations (*p* < 0.001), whereas Tiron alone did not produce a significant reduction (Figure 9D).

Colitis induction was also associated with a significant decrease in colonic SOD activity, from 0.047 ± 0.007 to 0.008 ± 0.001 (*p* < 0.001). Pretreatment with Tiron or with both NAC–Tiron combinations significantly restored SOD activity in colonic tissue (*p* < 0.01), whereas NAC alone did not significantly restore this enzymatic activity (Figure 9E).

Colitis induction also caused a significant decrease in colonic total thiol levels, from 21.385 ± 2.125 to 5.037 ± 1.085 mM/mg (*p* < 0.001). Pretreatment with NAC, Tiron, or the NAC–Tiron combination at the lower dose (100 mg/kg) significantly restored total thiol levels compared with the colitis group (*p* < 0.01). The higher dose of NAC–Tiron (200 mg/kg) also significantly restored colonic total thiol levels compared with the colitis group (*p* < 0.001) (Figure 9F). Similarly, colitic rats showed a marked decrease in colonic GSH levels, from 0.145 ± 0.015 to 0.031 ± 0.004 µM/mg (*p* < 0.001). Pretreatment with NAC, Tiron, or the higher dose of NAC–Tiron (200 mg/kg) significantly restored colonic GSH levels compared with the colitis group (*p* < 0.001). The lower dose of NAC–Tiron (100 mg/kg) partially increased GSH levels, although this effect did not reach statistical significance (Figure 9G).

### 2.6. NAC, Tiron and Their Combination Decreased Inflammatory and Oxidative Responses in Rat Colitis

**Figure 10:**
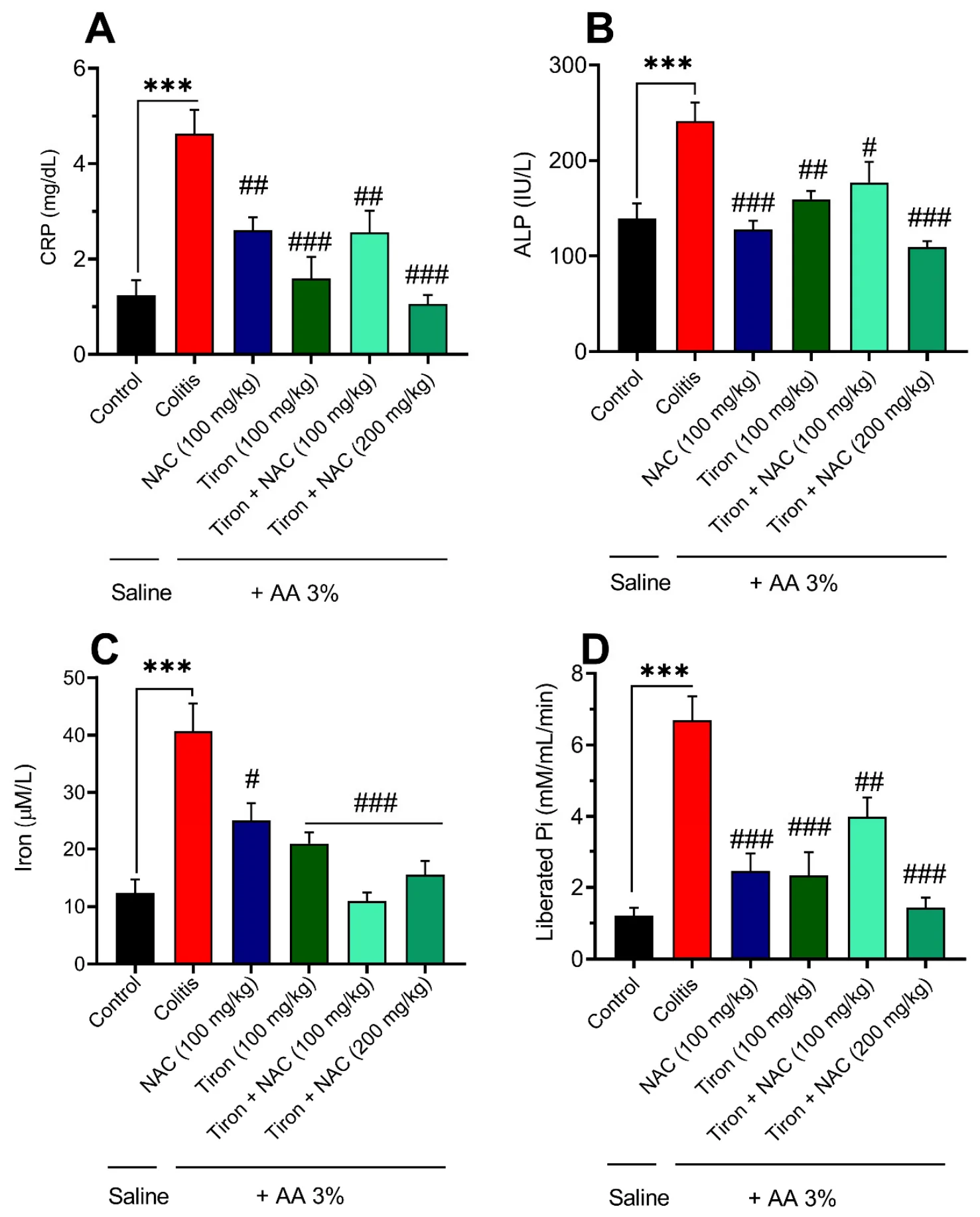
Effect of NAC, Tiron, and their combination on plasma and tissue inflammatory markers during colitis. Animals received intraperitoneal pretreatment for 14 days with NAC (100 mg/kg), Tiron (100 mg/kg), or NAC–Tiron at total doses of 100 mg/kg (50 mg/kg NAC plus 50 mg/kg Tiron) or 200 mg/kg (100 mg/kg NAC plus 100 mg/kg Tiron) before colitis induction. Blood pro-inflammatory markers, including CRP (**A**), ALP (**B**), and iron content (**C**). The dephosphorylation of glycerol-3-phosphate in colon homogenates, were determined by measuring the liberated inorganic phosphate (**D**). Results were expressed as mean ± S.E.M. for n = 6 rats per group. *** *p* < 0.001 compared with control rats, and # *p*  < 0.05, ## *p*  < 0.01, and ### *p* < 0.001 compared to rats with colitis.

C-reactive protein in rat plasma significantly increased during colitis, if compared with control animals (from 1.239 ± 0.3175 mg/dL to 4.629 ± 0.491 mg/dL, (*p* < 0.001). Interestingly, a significant decrease in this inflammatory mediator was detected in rats pretreated with NAC (2.6 ± 0.28 mg/dL, *p* < 0.01) or Tiron (1.596 ± 0.45 mg/dL, *p* < 0.001) (Figure 10A). The combination of Tiron and NAC significantly limited the rise in CRP at doses of 100 mg/kg (2.563 ± 0.442, *p* < 0.01) and 200 mg/kg (1.061 ± 0.184, *p* < 0.001) respect to colitis group. Plasma Alkaline phosphatase (ALP) activity was strongly increased after colitis induction (from 139.333 ± 15.827 to 241.389 ± 19.178, *p* < 0.001). Pretreatment with NAC prevented the increase in this enzymatic activity (127.875 ± 9.104, *p* < 0.001). In addition, Tiron clearly decreased ALP activity, albeit less significantly (to 159.500 ± 8.789, *p* < 0.01). The association NAC–Tiron at a dose of 100 mg/kg slightly but significantly decreased ALP activity (to 177.069 ± 21.468 IU/L, *p* < 0.05), while the higher dose also significantly decreased ALP activity (to 109.333 ± 6.337 IU/L, *p* < 0.001) (Figure 10B).

Colitis induced in rats by using AA is associated with a significant rise in plasma iron [46]. Also in our experiment, iron increase was significantly increased (from 12.364 ± 2.339 to 40.729 ± 4.796 mg/dL, *p* < 0.001), but was markedly decreased in rats receiving NAC (to 25.096 ± 3.009 mg/dL, *p* < 0.05) and Tiron (to 20.958 ± 1.969 mg/dL, *p* < 0.001). Pretreatment with NAC–Tiron, at 100 or 200 mg/Kg, strongly decreased plasma-free iron (*p* < 0.001) at values similar to those found in control rats (Figure 10C).

Furthermore, we found that colitis affects dephosphorylation of Glycerol-3-phosphate (G-3-P) in the colonic supernatant. This was evidenced by a significant increase in Pi in rats with colitis compared with control animals (from 1.213 ± 0.221 to 6.697 ± 0.666 Mm/mL/min; *p* < 0.001). However, pretreatment with NAC or Tiron triggered a significant decrease in this enzymatic activity compared to colitis rats (*p* < 0.001). The NAC–Tiron combination also decreased this enzymatic activity, at both 100 mg/Kg (to 3.997 ± 0.534, *p* < 0.01) and at 200 mg/Kg (to 1.348 ± 0.284, *p* < 0.001) (Figure 10D).

### 2.7. NAC, Tiron, and Their Combination Improve Liver Functions

**Figure 11:**
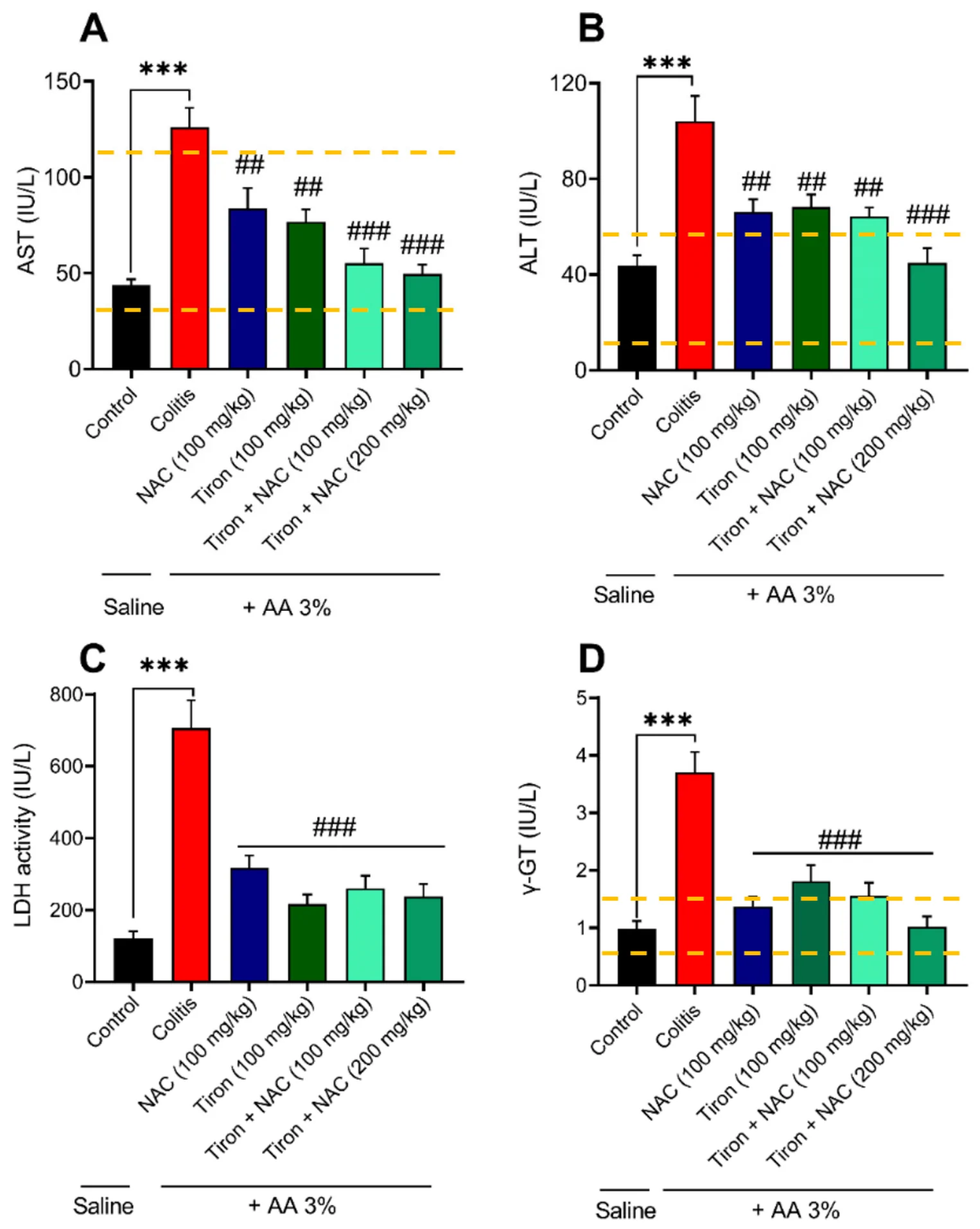
Effects of pretreatment with NAC, Tiron, and their combination (NAC–Tiron) liver biomarker (AST, ALT, γ-GT) and LDH activity during induced colitis. Rats received intraperitoneal pretreatment for 14 days with NAC (100 mg/kg), Tiron (100 mg/kg), or NAC–Tiron at total doses of 100 mg/kg (50 mg/kg NAC plus 50 mg/kg Tiron) or 200 mg/kg (100 mg/kg NAC plus 100 mg/kg Tiron) before colitis induction. AST (**A**), ALT (**B**), LDH (**C**), and γ-GT (**D**), have been determined in rat plasma. Results were presented as mean ± S.E.M. (n = 6 rats per group). The dashed orange lines represent the reference ranges, when available (U.C.L.A. suggested general range for adult male Wistar rats), for each individual parameter. *** *p* < 0.001 indicating a comparison to control rats ## *p* < 0.01, and ### *p* < 0.001 indicating a comparison to the colitis group.

Experimental colitis altered the liver metabolism via the augmentation of the AST activity (from 43.633 ± 3.202 IU/L to 126.117 ± 10.121 IU/L, *p* < 0.001). NAC or Tiron administration resulted in a significant decrease (*p* < 0.01) in AST compared to control group. NAC–Tiron combinations at both tested doses also significantly attenuated the AST increase (reductions to 55.183 ± 7.671 IU/L, *p* < 0.001 for 100 mg/Kg and 49.583 ± 4.834 IU/L, *p* < 0.001 for 200 mg/kg) (Figure 11A).

Also, ALT activity increased after colitis induction compared to control rats (from 43.750 ± 4.306 IU/L to 104.1 ± 10.6 IU/L, *p* < 0.001). However, NAC, Tiron or NAC-Tiron at 100 mg/kg significantly prevented the rise in ALT activity (*p* < 0.01). Interestingly, the co-treatment at the higher dose of 200 mg/kg significantly reduced ALT activity, bringing it back to a value within the reference range (44.800 ± 6.265 IU/L, *p* < 0.001) (Figure 11B). Plasma lactate dehydrogenase (LDH) activity is a well-established biomarker of systemic cytotoxicity and general tissue damage. It was found significantly increased in the colitis group, rising from 120.885 ± 20.018 IU/L in control rats to 705.344 ± 78.086 IU/L in colitic rats (*p* < 0.001). Pretreatment with NAC, Tiron, or their combination significantly attenuated the colitis-associated increase in LDH activity (Figure 11C). As shown in Figure 11D, colitis induction also caused a significant increase in plasma γ-GT, which rose from 9.870 ± 1.326 IU/L in control rats to 37.015 ± 3.570 IU/L in the colitis group (*p* < 0.001). This increase was significantly reduced in rats pretreated with NAC, Tiron and NAC–Tiron co-treatments. Metabolic profile analysis did not reveal major alterations among experimental groups (Supplementary Figure S1). In rats treated with NAC, Tiron, or NAC–Tiron, resting blood glucose, triglycerides, cholesterol, glycerol, and creatinine levels were reduced compared with the colitis group. Although some of these changes reached statistical significance, the values remained within the expected reference ranges in all experimental groups.

## 3. Discussion

The present study investigated NAC, Tiron, and their combination through complementary in silico, in vitro, and in vivo approaches to characterize their antioxidant and protective effects in acetic acid-induced colitis. NAC and Tiron are chemically defined antioxidant molecules, but they differ in their chemical nature and biological profile. NAC is an N-acetylated derivative of the naturally occurring amino acid L-cysteine and acts mainly as a thiol-containing antioxidant and glutathione precursor, whereas Tiron is a synthetic water-soluble catechol derivative known for its metal-chelating and radical-scavenging properties. The rationale for combining NAC and Tiron was based on their distinct chemical properties potentially relevant to redox control. NAC is a thiol-containing compound and a precursor of glutathione synthesis [24], whereas Tiron is a water-soluble catechol derivative with radical-scavenging and metal-chelating properties [34]. These characteristics provided a basis for evaluating whether both compounds could retain protective activity when administered together. However, the molecular pathways through which the combined preparation acted were not directly measured, and the present findings do not establish complementary mechanisms or pharmacological synergy.

In silico approaches are increasingly used as exploratory tools to evaluate molecular complementarity and to support the selection of compounds for subsequent in vitro and in vivo testing. In the present study, ligand–ligand docking was used to assess whether NAC and Tiron could establish plausible non-covalent interactions. This analysis was based on the chemical structures and functional groups of the two molecules. Tiron contains hydroxyl and sulfonic groups attached to an aromatic benzene ring, structural features that contribute to its polarity, metal-chelating capacity, and radical-scavenging potential [35,47]. NAC, conversely, is an N-acetylated derivative of L-cysteine whose thiol, amide, and carboxyl groups contribute to its antioxidant reactivity and capacity to participate in polar interactions [48].

The docking simulations suggested that NAC and Tiron may interact through hydrogen bonds, electrostatic contacts, and van der Waals interactions involving the polar functional groups of both molecules. The lower predicted binding energy observed for the NAC–Tiron complex compared with the NAC–periodic acid complex, together with the more favorable RMSD value, supports the existence of a more defined interaction geometry between NAC and Tiron under the computational conditions used. These findings are consistent with the high polarity of Tiron and the ability of NAC to engage in hydrogen bonding and electrostatic interactions.

Although these ligand–ligand docking data should be interpreted with caution because no macromolecular target was included and no experimental validation of complex formation was performed, the predicted molecular complementarity suggests that NAC and Tiron can coexist in a spatial arrangement that allows non-covalent interactions between their polar functional groups while preserving the structural features responsible for their antioxidant activity. In particular, the docking model suggests that the thiol-containing region of NAC and the hydroxyl/sulfonate groups of Tiron may remain available to participate in redox-related reactions. This supports the use of the combined preparation in subsequent functional assays, not as evidence of a stable pharmacological complex, but as a preliminary indication that the two molecules are chemically compatible and may retain complementary antioxidant properties when tested together. The in vitro antioxidant assays further supported the redox-modulating properties of NAC, Tiron, and their combination. The three preparations were able to scavenge ABTS•+ and DPPH• radicals, chelate Fe^2+^, and display ferric-reducing activity, although their relative efficacy differed according to the assay used. These results are consistent with previous evidence showing that Tiron can chelate transition metals and participate in redox reactions involving metal ions [35], as well as with reports confirming the antioxidant and acellular free-radical-scavenging properties of NAC [49]. The structural features of Tiron, including hydroxyl and sulfonic groups on the aromatic ring, may explain its marked iron-chelating activity, whereas the thiol group of NAC likely contributes to its radical-scavenging and reducing properties [47,48]. The relative efficacy of the preparations differed according to the assay examined. The NAC–Tiron combination showed particularly strong DPPH radical-scavenging activity, with a lower IC50 than NAC, Tiron, and BHT. In contrast, NAC, Tiron, and NAC–Tiron showed comparable IC50 values in the ABTS assay, whereas Tiron alone displayed the strongest iron-chelating activity. The combination also retained anti-denaturation activity but did not clearly outperform NAC in this assay. Therefore, the in vitro findings support greater activity of NAC–Tiron in selected experimental conditions, particularly DPPH radical scavenging, rather than a uniformly broader or superior antioxidant profile. Because no formal pharmacological interaction analysis was performed, these results cannot be interpreted as evidence of synergy.

The antioxidant profile observed in the acellular assays is also relevant considering previous studies showing that NAC and Tiron may influence redox-sensitive inflammatory pathways. NAC has been reported to modulate Nrf-2- and MAPK-related signaling and to attenuate inflammatory responses by affecting matrix metalloproteinase activity, MAPK phosphorylation, and intercellular adhesion molecule-1 regulation [27,28]. These effects are consistent with its role as a thiol-containing antioxidant and glutathione precursor, which supports intracellular redox homeostasis [24,25,26]. Tiron, in turn, has been reported to limit mitochondrial reactive oxygen species, superoxide anion, peroxynitrite, and hydrogen peroxide production [37,38,41], and to modulate inflammatory and antioxidant pathways, including NF-κB, Nrf-2, NADPH oxidase-related signaling, and TNF-α release [39,40,50]. Therefore, although these molecular pathways were not directly investigated in the present study, the observed antioxidant properties of NAC, Tiron, and their combination are consistent with mechanisms previously described for these compounds. In this context, the protein-denaturation assay provided a preliminary acellular indication of anti-denaturation activity. NAC showed marked activity, with an IC50 close to that of diclofenac, whereas Tiron was less effective. The NAC–Tiron combination retained anti-denaturation activity, with an IC50 of 2.114 ± 0.563 mg/mL. However, this value was numerically higher than those obtained for NAC and diclofenac, and the combination did not clearly outperform NAC alone in this assay. These findings should therefore be interpreted cautiously. Intrarectal administration of acetic acid induced marked macroscopic and histological colonic damage, including redness, thickening of the colon, increased wet colon weight, epithelial disruption, inflammatory cell infiltration, and edema. These alterations are consistent with previous descriptions of acetic acid-induced colitis as a reproducible model of acute mucosal injury characterized by epithelial damage, inflammatory infiltration, and oxidative stress [51,52,53]. Pretreatment with NAC and Tiron alone attenuated these pathological changes as already observed in previous studies [23,50] but the combined treatment also preserved colonic architecture and reduced inflammatory cell accumulation, edema, and epithelial damage. Histological scoring confirmed that all pretreatments significantly reduced tissue damage compared with the colitis group (Figure 8B). Although selected pairwise comparisons among the pretreated groups reached statistical significance, these differences were confined to the histological endpoint and should not be generalized as evidence of consistent superiority of NAC–Tiron across the other in vivo outcomes.

Oxidative stress represents a central component of acetic acid-induced colonic injury. In the present study, colitis was associated with a marked increase in lipid peroxidation and protein oxidation, as indicated by elevated MDA and protein carbonyl levels in plasma and/or colonic tissue. These findings are consistent with previous evidence showing that experimental colitis promotes excessive free-radical production, myeloperoxidase-related oxidative injury, lipid peroxidation, and protein oxidation in inflamed intestinal tissues [54,55,56,57,58]. In our study, NAC and Tiron reduced MDA accumulation, in line with earlier studies reporting antioxidant protection by NAC or Tiron in experimental models of intestinal inflammation and oxidative injury [23,31,50,59]. In addition to reducing pro-oxidant markers, NAC, Tiron, and their combination helped preserve endogenous antioxidant defenses. Acetic acid-induced colitis decreased colonic SOD activity, total thiols, and GSH levels, confirming a disruption of redox homeostasis. NAC may contribute to the restoration of this balance through its role as a thiol-containing antioxidant and glutathione precursor, whereas Tiron may reduce the oxidative burden by scavenging reactive species and limiting metal-associated oxidative reactions [36,45,60,61]. In this context, NAC–Tiron reduced oxidative damage and supported the recovery of several antioxidant defenses relative to the colitis group. However, the combination did not consistently outperform the individual treatments across these endpoints.

These findings are particularly relevant because oxidative damage and antioxidant depletion are closely linked to mucosal injury and inflammatory amplification in colitis. However, although NAC–Tiron improved several oxidative stress endpoints relative to the colitis group, these data should not be interpreted as evidence of pharmacological synergy or consistent superiority over the individual treatments. Rather, they indicate that NAC–Tiron co-administration attenuated several indices of colitis-associated redox imbalance under the present experimental conditions. The modulation of inflammatory and iron-related biochemical markers further supports the protective profile of NAC, Tiron, and their combination. In the present study, acetic acid-induced colitis increased plasma CRP, ALP activity, and free iron levels, indicating systemic inflammatory activation and a pro-oxidant biochemical environment. CRP is a well-established marker of inflammatory burden in IBD patients, whereas ALP has been associated with intestinal inflammation and mucosal responses [62,63]. The reduction in CRP and ALP after pretreatment with NAC, Tiron, or NAC–Tiron therefore supports the ability of these compounds to attenuate colitis-associated inflammatory alterations.

The increase in plasma free iron is also relevant in the context of oxidative injury. Free or poorly liganded iron can amplify oxidative damage by promoting iron-dependent redox reactions and excessive free-radical generation, thereby contributing to tissue injury during intestinal inflammation [64,65,66]. In this study, NAC, Tiron, and both NAC–Tiron combination groups reduced plasma free iron levels in colitic rats. This effect is consistent with the in vitro iron-chelating activity observed for the tested compounds, particularly Tiron, and may contribute to limiting iron-associated oxidative reactions during colitis.

Taken together, the reductions in free iron, CRP, and ALP provide a coherent link between the acellular antioxidant findings and the in vivo biochemical response. These changes support the biological relevance of NAC–Tiron co-administration in attenuating both inflammatory and pro-oxidant disturbances in this model, although they do not establish a specific mechanism of action or consistent superiority over the individual treatments. Nevertheless, because markers of ferroptosis, cytokine production, and inflammatory signaling pathways were not directly measured, these findings should be interpreted as evidence of reduced inflammatory and iron-related oxidative burden rather than as proof of specific pathway inhibition or ferroptosis prevention.

In parallel with the intestinal endpoints, different plasma biochemical parameters were assessed to verify the systemic profile of the treatments. In the present study, acetic acid-induced colitis was associated with marked increases in some enzymatic markers, particularly transaminases, LDH, and γ-GT, suggesting a systemic biochemical response to acute intestinal injury. Overall, pretreatment with NAC, Tiron, or NAC–Tiron attenuated several colitis-associated alterations in systemic biochemical markers. Although some metabolic parameters differed among experimental groups, glucose, cholesterol, and creatinine values remained within the expected physiological ranges. These findings show no evident worsening of the measured biochemical profile during the 14-day pretreatment period, but they provide only limited information on short-term biochemical tolerability and do not establish comprehensive safety. However, no dedicated pharmacokinetic or drug–drug interaction studies were performed for the NAC–Tiron combination. Therefore, the present data do not allow conclusions regarding mutual effects on absorption, distribution, metabolism, or elimination. From a pharmacodynamic perspective, NAC may influence thiol and glutathione homeostasis [24], whereas Tiron has radical-scavenging and metal-chelating properties [34]. Their co-administration may produce combined redox effects, but it could also alter redox balance or metal availability in a dose- and context-dependent manner. Potential effects on essential-metal homeostasis and organ-specific toxicity were not assessed and require direct experimental investigation. The absence of evident worsening of the biochemical parameters measured during the 14-day pretreatment period supports only limited short-term biochemical tolerability under the present conditions and should not be interpreted as evidence of comprehensive safety. Future studies should include pharmacokinetic measurements, essential-metal homeostasis, hematological and organ-specific toxicity endpoints, and longer treatment periods. The present study has several limitations. First, the absence of a standard pharmacological comparator prevents direct benchmarking of NAC–Tiron against mesalamine, sulfasalazine, or other established ulcerative colitis treatments. Second, only one fixed 1:1 mass ratio and two total combination doses were examined. Because matched groups receiving 50 mg/kg NAC or 50 mg/kg Tiron alone were not included, the effect of the 50 mg/kg NAC plus 50 mg/kg Tiron combination cannot be compared with the effects of the same component doses administered separately. Similarly, any greater effect of the 100 mg/kg NAC plus 100 mg/kg Tiron combination may reflect the higher total administered dose. In the absence of multiple dose ratios, a factorial dose–response design, and a formal reference model of additivity, the present results cannot distinguish independent, additive, or synergistic effects [67].

In addition, the study used a short-term preventive protocol in a single acute model, with the compounds administered for 14 days before colitis induction and the outcomes assessed 24 h after acetic acid administration. The findings therefore cannot be directly extrapolated to the treatment of established or chronic colitis. The use of only male rats and six animals per group also limits the generalizability of the results. Furthermore, pro-inflammatory cytokines, myeloperoxidase activity, inflammatory signaling pathways, pharmacokinetics, drug–drug interactions, and dedicated toxicity endpoints were not directly assessed. Finally, the ligand–ligand docking and protein-denaturation assays should be regarded as exploratory approaches rather than direct evidence of molecular interaction or in vivo anti-inflammatory mechanisms. Further studies should include standard pharmacological comparators, matched single-agent doses, multiple combination ratios, mechanistic endpoints, and longer-term safety assessments.

## 4. Materials and Methods

### 4.1. Chemicals and Reagents

N-acetylcysteine, Tiron (disodium 4,5-dihydroxy-1,3-benzenedisulfonate), periodic acid, ethylenediaminetetraacetic acid (EDTA), Trolox, BHT (butylated hydroxytoluene), ascorbic acid, ABTS (2,2′-azino-bis(3-ethylbenzothiazoline-6-sulfonic acid)), disodium hydrogen phosphate (Na_2_HPO_4_), DPPH (2,2-diphenyl-1-picrylhydrazyl), phosphate-buffered saline (PBS), monopotassium phosphate (KH_2_PO_4_), sodium phosphate dibasic (NaHPO_4_), magnesium chloride (MgCl_2_), sodium chloride (NaCl), sulphuric acid, diclofenac sodium salt, sodium carbonate (Na_2_CO_3_), potassium chloride (KCl), triethanolamine, ammonium acetate, and sn-glycerol 3-phosphate bis(cyclohexylammonium) salt were obtained from Sigma Aldrich-Merck (St. Louis, MO, USA). Sodium chloride (0.9%) was obtained from Infomed Pharma (London, UK). Acetic acid was purchased from VWR International (Radnor, PA, USA). Hydrochloric acid (HCl) from Thermo Fisher Scientific (Waltham, MA, USA). Aspartate aminotransferase (Reference 10032), alanine aminotransferase (Reference 11022), C-reactive protein kit (Reference 41012), lactate dehydrogenase (Reference 12029), γ-glutamyl transferase (γ-GT) (Reference 09029), glucose content (Reference 26040), cholesterol level (Reference 21014), triglycerides (Reference 21038), creatinine (Reference 25012), alkaline phosphatase (Reference 13026), and free iron content (Reference 20061) were purchased from the Biomaghreb company (Tunis, Tunisia). Paraffin, hematoxylin, eosin, ethanol, and ethanol 95%, methanol, and formol were provided by VWR International (Radnor, PA, USA).

### 4.2. In Silico Molecular Analysis of NAC–Tiron Interactions

Molecular structures of NAC and Tiron in SDF format were obtained from the PubChem database (CIDs 12035 and 5111182, respectively). For Tiron, counter-ions were suppressed to minimize artefacts during docking simulations. The 3D structure of periodic acid (H_5_IO_6_) was manually generated and the chemical connectivity validated before saving in PDB format for subsequent analysis. These molecules were then converted into 3D models and geometry-preformed using Avogadro (version 1.2) with the MMFF94 force field. Furthermore, the physicochemical properties of these molecules were obtained from the PubChem database (NCBI) and verified by structural inspection. Hydrogen-bond donors and acceptors were assigned based on functional group chemistry, according to standard definitions. The molecular weights and chemical formulas of the compounds were obtained from PubChem, while key structural features and interaction behavior were deduced from molecular topology and functional groups to support docking analysis. Molecular docking assays were carried out via the use of AutoDock Vina (version 1.2.5). An exploratory ligand–ligand docking strategy was adopted due to the absence of a classical macromolecular target. In this approach, one molecule was treated as a ligand and the second as a pseudo-receptor to assess the physicochemical interactions and complementarity between them. AutoDock Tools (version 1.5.7) was applied to convert all PDB files to PDBQT format, add polar hydrogens, and assign Gasteiger charges according to the standard AutoDock preparation protocols. Docking was performed using blind docking with grid boxes covering the entire pseudo-receptor (molecule 1), default AutoDock Vina parameters, and an exhaustiveness value of 8. Docking poses were ranked by the predicted binding affinity (kcal/mol) provided by AutoDock Vina, and the binding energy values were used only for comparative and qualitative analysis. The docked complexes were visualized using UCSF Chimera (version 1.16). The most favorable poses were characterized by measuring intermolecular distances, with hydrogen bonds and polar interactions identified using standard geometric criteria.

### 4.3. Antioxidant and Anti-Inflammatory Evaluation of NAC, Tiron and Their Combination

The antioxidant properties of NAC and Tiron, as well as their combined form (NAC–Tiron), were assessed using various acellular systems. This includes the ABTS test [68], which was monitored over a fixed period of 40 min and kinetically every 5 min at 734 nm, using ascorbic acid as a standard agent. The technique described by [69] was utilized with slight modifications to assess the efficacy of these molecules in scavenging DPPH^•−^ over 40 min and kinetically every 5 min at 517 nm, using BHT as the standard antioxidant agent. Additionally, the ability of these molecules to chelate free iron was determined at 562 nm, according to the technique described by [70]. Furthermore, the capacity of NAC and Tiron molecules, as well as their combined form (NAC–Tiron), to transform Fe^2+^ to Fe^3+^ was examined using the colorimetric method of [71]. This was determined at 700 nm using Trolox (standard reagent) and the tested molecules prepared at the same concentration range from 0 to 160 µg/mL. The anti-inflammatory properties of NAC and Tiron, and their combination (NAC–Tiron), were evaluated by assessing their ability to limit the protein denaturation, this was achieved using the technique previously described by [72], with minor modifications. Increasing concentrations of NAC and Tiron (0, 0.5, 1, 2, and 4 mg/mL) were used, as well as the prepared NAC–Tiron solution at the same concentrations. A buffered solution was prepared using NaCl (0.136 M), KCl (2.68 mM), NaHPO_4_ (10.1 mM), and KH_2_PO_4_ (1.76 mM), and the pH was adjusted to 6.3 using a 0.1 N HCl solution. Briefly, all preparations were made using the buffered solution, and then a volume of 100 µL of each concentration was adjusted with BSA (1%) for a total volume of 1 mL per tube. All preparations were then incubated in a water bath (37 °C) for 20 min before being heated at 90 °C for 3 min and cooled. Absorbance was then monitored at 660 nm. All assays were performed in triplicate and calculated using the formula published in the study of [72]. A standard curve was created using the diclofenac reagent prepared in dimethyl sulfoxide at the same concentrations.

### 4.4. Animal Model and Experimental Protocol

Thirty-six male Wistar rats (140 ± 20 g) were purchased from the Pasteur Institute of Tunis (Tunisia). All rats were randomly assigned and kept under controlled conditions, including a stable room temperature (22 ± 2 °C), humidity (60–70%), and unrestricted access to water and rodent feed obtained from El Badr Society (Utique zone, Route of Tunisia). After the acclimation phase (14 days), rats were divided into six experimental groups (n = 6). Control and colitis groups received a daily intraperitoneal injection of PBS for 14 days. The single-agent groups received NAC or Tiron at 100 mg/kg. In the combination groups, NAC and Tiron were administered at a fixed 1:1 mass ratio: 50 mg/kg NAC plus 50 mg/kg Tiron for a total dose of 100 mg/kg, or 100 mg/kg NAC plus 100 mg/kg Tiron for a total dose of 200 mg/kg. The lower-total-dose combination was included as an exploratory assessment of co-administration at reduced individual doses, whereas the higher-total-dose combination contained the same dose of each compound used in the corresponding monotherapy groups. The NAC regimen was informed by a previous rat study in which NAC was administered at 100 mg/kg/day i.p. for 14 days [73], whereas the 100 mg/kg Tiron dose was selected on the basis of previous repeated-dose i.p. administration in rats [40]. However, the fixed 1:1 mass ratio and the use of these regimens as preventive co-treatment in the present acetic acid-induced colitis model were exploratory choices and were not intended to identify an optimal combination ratio or to demonstrate pharmacological synergy.

After 14 days, all groups, excluding the control, were administered with AA 3% i.r. (5 mL/Kg) to induce colitis [54]. Control group received i.r. NaCl 0.9% (5 mL/Kg). Then, 24 h after colitis induction, all animals were anesthetized and sacrificed by cervical dislocation [74]. During the experiment, body weights of rats were daily recorded. All experiments were conducted in accordance with the European Community Council Directive 2024/1262 and with rigorous application of the guidelines for the care and use of laboratory animals and the recommendations of the European Convention for the Protection and Use of Vertebrate Animals. Furthermore, the ethical committee of the Tunisian Association of Laboratory Animal Science approved the release of all experiments under the approval number No. 0134/2024 ATSAL.

### 4.5. Sample Collection and Tissue Processing

Blood samples were collected by cardiac puncture before euthanasia by cervical dislocation and stored into heparinized tubes. The tubes were centrifuged (4000 rpm, 4 °C, for 15 min); the plasma was recovered and aliquoted into sterile Eppendorf tubes (Hamburg, Germany, 3 tubes per rat), a stored at −25 °C until analysis. Liver, spleen, and colon were harvested, rinsed, and weighed (Biobase BA2204 balance, BIOBASE Group, Jinan, Shandong, China, accuracy 0.1 mg) before being stored at −25 °C. Moreover, to assess macroscopic markers of colitis, colonic tissues were weighed, and their length was measured to calculate the wet colon weight [55]. Additionally, distal colonic tissues were sectioned into small fragments, then thoroughly homogenized by using an electric homogenizer (T 10 basic Ultra-Turrax, IKA, Staufen im Breisgau, Germany) to prepare a 1:10 (*w*/*v*) sample in a homogenizing buffer (cold PBS, 0.1 M, pH 7.4) for oxidative stress assessment. All homogenization steps were performed in an ice bath. The resulting samples were centrifuged (9000 rpm, 4 °C) for 30 min (Eppendorf 5415R, Leipzig, Germany), and the obtained supernatants were collected and stored at −25 °C.

### 4.6. Histological Evaluation of the Distal Colonic Tissues

The colonic tissue of the rats (distal region) was rapidly excised and immediately placed in amber containers containing a 10% formalin solution, which were then kept at room temperature for 24 h. Each colonic fragment was then submerged in various ethanol preparations before being encased in paraffin, sectioned into 5 µm parts, and stained using two different methods: hematoxylin and eosin (H&E) and alcian blue (AB). Micrographs of the obtained slides were then taken at two different magnifications using a Carl Zeiss light microscope (Jena, Germany). Furthermore, the method of Geboes et al. [75] was used to calculate the histological score based on the evaluation of the obtained micrographs performed by a trained physiologist in blind (assigning from 0 to 3 points for each specific criteria). The final score is based on 4 different criteria including the degree of alterations of colonic microstructure, the presence or absence of inflammatory cells, the crypt damage and the presence of edema.

### 4.7. Antioxidant Assays in Tissue Homogenates

Pro-oxidant markers, including lipid peroxidation [76] and protein carbonylation [77], were examined in tissue homogenates. The activity of superoxide dismutase (SOD) [78], total thiol [79] and reduced glutathione (GSH) [80] were determined to evaluate antioxidant status. These parameters were expressed per milligram of total protein in the colonic tissue, as determined using the Folin–Ciocalteu technique [81].

### 4.8. Inflammatory Marker Assessment

Inflammatory responses during colitis were assessed by using the CRP kit (Reference 41012 Biomaghreb, Tunis, Tunisia), the enzymatic activity of alkaline phosphatase (ALP, Reference 13026 Biomaghreb, Tunis, Tunisia), and the determination of free iron content (Reference 20061 Biomaghreb, Tunis, Tunisia). Those tests were performed in the collected plasma of rats and examined following the instructions mentioned in the commercialized kits. Glycerol 3-phosphate phosphatase activity was examined in colonic tissue samples by determining the amount of inorganic phosphate released from sn-glycerol-3-phosphate using the technique of Ames [82]. In brief, each colonic tissue sample was sonicated vigorously for 10 s at 4 °C using a digital sonifier (Branson, Brookfield, CT, USA). Then, 50 µL of each sample was mixed with an equal volume of triethanolamine (0.01 M, pH 7.6). After this step, NaCl (4 M) and MgCl_2_ (0.1 M) solutions were added (15 µL from each reagent), followed by 120 µL of sn-glycerol 3-phosphate bis(cyclohexylammonium) salt (25 mM). The reaction was initiated by adding the working solution (700 µL), which was formed by ascorbic acid (10%) and ammonium molybdate (0.42%, dissolved in 1 N sulphuric acid) and incubated for one minute before measuring the absorbance at 820 nm. Results were calculated using a KH_2_PO_4_ standard curve prepared under the same conditions.

### 4.9. Systemic Biochemical and Metabolic Profile Assessment

To examine liver function, enzymatic activities of lactate dehydrogenase (LDH) and transaminases, including aspartate aminotransferase (AST) and alanine aminotransferase (ALT), were measured at 340 nm using commercial kits (References 12029, 10032, and 11022 Biomaghreb, Tunis, Tunisia). Furthermore, the γ-GT was examined in plasma at 450 nm using a commercial kit (Reference 09029, Biomaghreb, Tunis, Tunisia). The glucose content (Reference 26040 Biomaghreb, Tunis, Tunisia), cholesterol levels (Reference 21014 Biomaghreb, Tunis, Tunisia), and triglyceride (TGC) content (Reference 21038 Biomaghreb, Tunis, Tunisia) were assessed using spectrophotometry at an absorbance wavelength of 505 nm. Furthermore, plasma creatinine was measured at 492 nm using a commercial biochemical kit (Reference 25012). Additionally, the colonic glycerol content was examined using the previously described protocol of Bompelly and Skaff [83], and the absorbance was determined at 410 nm and compared with a glycerol standard curve.

### 4.10. Data Analysis

Quantitative results are expressed as mean ± standard error of the mean (SEM). For in vitro assays, data were obtained from three independent experiments, whereas in vivo experiments included six animals per experimental group. Statistical analyses were performed using GraphPad Prism 8 software (GraphPad Software, San Diego, CA, USA). Comparisons among multiple independent experimental groups were performed using one-way ANOVA followed by Tukey’s multiple comparisons test. For analyses involving two experimental factors, such as treatment and concentration or treatment and time, two-way ANOVA followed by Šidák’s multiple comparisons test was applied. Repeated measurements, when applicable, were analyzed using repeated-measures two-way ANOVA followed by Šidák’s multiple comparisons test. Half-maximal inhibitory concentration (IC_50_) values were estimated from dose–response curves by nonlinear regression analysis. Differences were considered statistically significant at *p* ≤ 0.05.

## 5. Conclusions

In summary, the present study provides an integrated evaluation of NAC, Tiron, and their combination using in silico, in vitro, and in vivo approaches. Ligand–ligand docking suggested possible physicochemical compatibility between NAC and Tiron through non-covalent interactions. In vitro, NAC, Tiron, and their combination showed antioxidant activity by scavenging free radicals, modulating iron-related redox reactions, and limiting protein denaturation.

In the acetic acid-induced colitis model, 14-day pretreatment with NAC, Tiron, or NAC–Tiron attenuated macroscopic and histological colonic damage, reduced inflammatory cell infiltration and edema, decreased oxidative damage markers in plasma and colonic tissue, and helped preserve endogenous antioxidant defences, including SOD activity, thiol levels, and GSH content. The treatments also attenuated the increase in CRP and free iron associated with colitis and did not induce evident worsening of the systemic biochemical profile under the present experimental conditions.

Overall, the novelty of the present study lies in the exploratory assessment of NAC and Tiron administered together at a fixed 1:1 mass ratio in acetic acid-induced colitis, rather than in the individual effects of either compound. The combination showed enhanced activity in selected endpoints, particularly DPPH radical scavenging, but did not consistently produce greater effects than the individual treatments across the other in vitro and in vivo outcomes. Because no standard pharmacological comparator, matched low-dose monotherapy groups, or formal interaction analysis was included, these results should not be interpreted as evidence of pharmacological synergy, superiority over established therapies, or clinical efficacy. Further studies using multiple dose ratios, appropriate standard comparators, and dedicated pharmacological interaction analyses are required.

## Figures and Tables

**Table 1:** Physicochemical properties of NAC, Tiron, and periodic acid relevant for molecular docking and interaction analysis.

Molecule	Formula	Mw	H Bond		Molecular Structure
			Donors	Acceptors	
NAC	C_5_H_9_NO_3_S	163.19	2	4	Thiol group, amide, Carboxyl, benzene ring, Sulfonate, hydroxyl
Tiron	C_6_H_4_O_8_S_2_^2−^	300.26	2	8	Benzene ring, Sulfonate hydroxyls
Periodic acid	H_5_IO_6_	227.94	5	6	Central iodine, Multiple hydroxyls

**Table 2:** Docking scores and pose stability parameters for NAC interactions with Tiron and periodic acid.

Interaction	NAC–Tiron	NAC–Periodic Acid
Docking score	−25.18	−14.30
Confidence score	0.078	0.062
Ligand RMSD (Ångström)	7.3	28.55

## Data Availability

The original contributions presented in this study are included in the article/Supplementary Material. Further inquiries can be directed to the corresponding author.

## Supplementary Materials

The following supporting information can be downloaded at https://www.mdpi.com/article/10.3390/ijms27167146/s1.

## Abbreviations

The following abbreviations are used in this manuscript:

AA	Acetic acid
ABTS	2,2′-azino-bis(3-ethylbenzothiazoline-6-sulfonic acid)
ALP	Alkaline phosphatase
CD	Crohn’s disease
CRP	C-reactive protein
DPPH	2,2-diphenyl-1-picrylhydrazyl
EMU	External mucosa
FRs	Free radicals
G-3-P	Glycerol-3-phosphate
H_5_IO_6_	Periodic acid
HCl	Hydrochloric acid
IBD	Inflammatory bowel disease
i.r.	Intrarectal
i.p	Intraperitoneal
IL	Interleukin
K_2_S_2_O_8_	Potassium persulfate
LP	Lamina propria
MAPK	Mitogen-activated protein kinase
MMU	Muscularis mucosa
MU	Mucosa
Na_2_CO_3_	Sodium carbonate
NAC	N-acetylcysteine
NADPH	Nicotinamide adenine dinucleotide phosphate
NaHCO_3_	Sodium bicarbonate
NF-κB	Nuclear factor-kappa B
Nrf-2	Nuclear factor erythroid-2-related factor 2
PBS	Phosphate-buffered saline
ROS	Reactive oxygen species
SOD	Superoxide dismutase
SMU	Submuscularis mucosa
TGC	Triglycerides
TNF-α	Tumor necrosis factor-α
UC	Ulcerative colitis
