# Supplementary material for: N-Acetylcysteine, Tiron, and Their Combination: In Vitro Antioxidant and Anti-Inflammatory Activities and Their Protective Effect in a Rat Model of Acetic Acid-Induced Ulcerative Colitis
Source: Int J Mol Sci. 2026 Aug 10;27(16):7146. doi: 10.3390/ijms27167146 (PMC13513142; doi:10.3390/ijms27167146)
Supplement: Supplementary file 1 [file ijms-27-07146-s001.zip › ijms-4452109-supplementary.pdf]

**Supplementary Figure S1.** NAC, Tiron and their combination modify metabolic markers during colitis

Glucose levels in plasma (**Supplementary Figure S1A**) shows a significant augmentation ($p < 0.001$) in rats with colitis from 93.899 ± 3.726 mg/dL to 128.988 ± 6.216 mg/dL. Pretreatment with NAC decreased glycaemia (110.042 ± 3.917 mg/dL, $p < 0.05$) as well as Tiron pretreatment (97.082 ± 1.824 mg/dL, $p < 0.001$). Also, the co-administration with these molecules lowered ($p < 0.001$) plasma glucose levels compared to the colitis group. Nevertheless, glucose levels remained within the reference ranges in all experimental groups.

Rats with colitis are also marked by a significant augmentation ($p < 0.001$) in triglycerides (from 0.262 ± 0.097 to 1.602 ± 0.181 mM/L) and total cholesterol (from 0,602 ± 0.054 to 0.956 ± 0.069 g/L). Both NAC and Tiron pretreatment lowers triglycerides ($p < 0.001$) and cholesterol ($p < 0.01$) and a similar significant decrease ($p < 0.001$) in these two lipid markers was observed in rats receiving NAC–Tiron co-administration at 100 and 200 mg/Kg. Even cholesterol and triglycerides levels in plasma remained within the reference ranges in all experimental groups (**Supplementary Figure S1B** and **S1C**). Glycerol content significantly increased in colitis (from 0.037 ± 0.006 to 0.169 ± 0.018 mM/mL). Pretreatment with NAC, Tiron, or their association ($p < 0.001$) to prevent the rise of this lipid marker that remained within the reference ranges in all experimental groups. Colitis had some effects on renal homeostasis increasing plasma creatinine (from 0.554 ± 0.134 m to 3.785 ± 0.382 mg/L, values within the reference ranges). Pretreatment with NAC, Tiron and their association significantly stabilized plasma creatinine at lower levels, compared to colitis rats **(Supplementary Figure S1E)**.

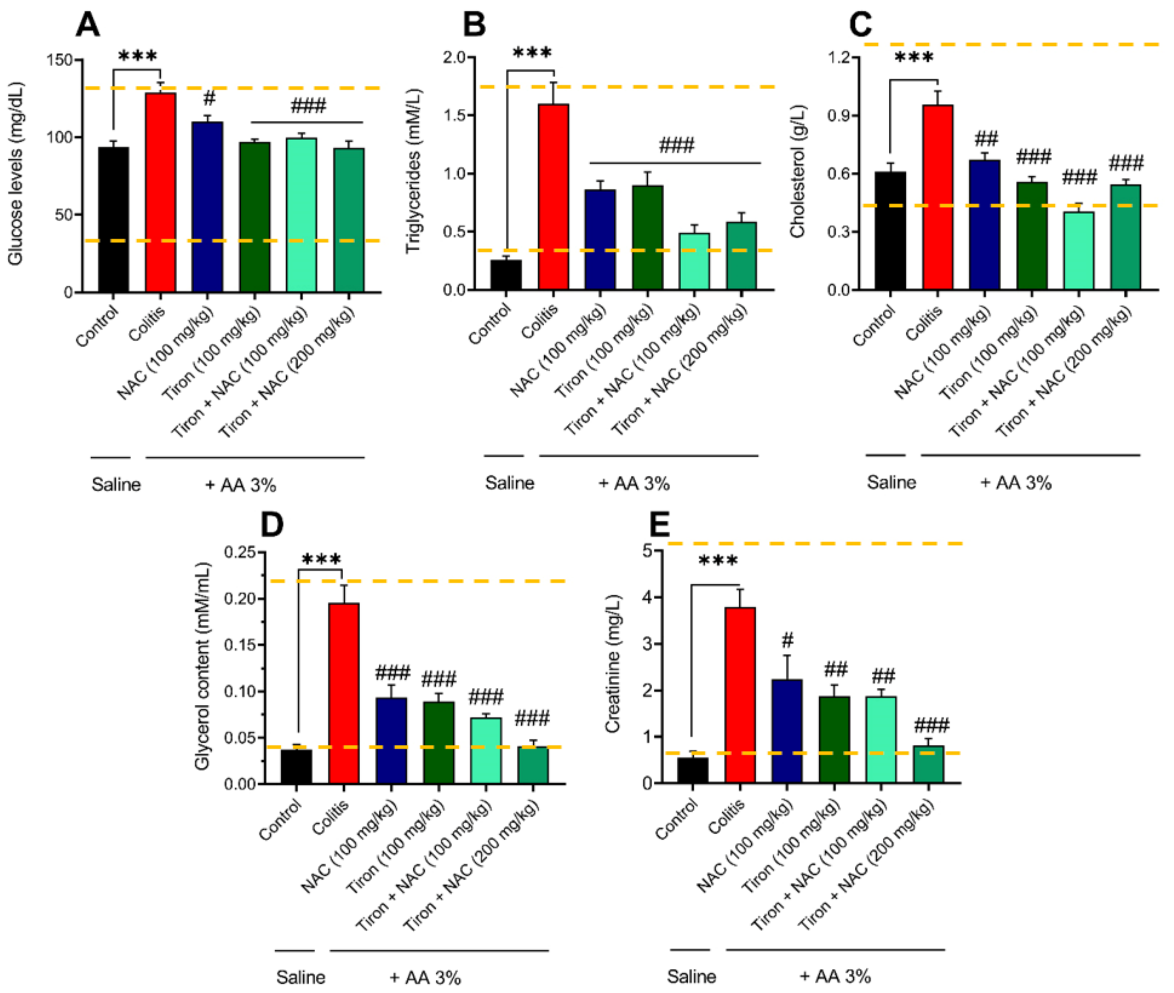


**Supplementary Figure S1:** Effect of NAC, Tiron, and their combination on markers of energetic metabolism during colitis induced by AA 3% in rats. Animals were treated with NAC (100 mg/kg), Tiron (100 mg/kg), and the combination of these molecules at two doses (100 and 200 mg/kg) for two weeks via the *i.p.* route. AA 3% was then administered to all rats except the control group, which received saline. Plasma metabolic markers including glucose (**A**), triglycerides (**B**), cholesterol (**C**), glycerol content (**D**) and creatinine (**E**), were measured, and results are expressed as mean ± S.E.M (n = 6). The dashed orange lines represent the reference ranges (U.C.L.A. suggested general range for adult male Wistar rats) for each individual parameter. *** $p < 0.001$ compared with control animals, and # $p < 0.05$, ## $p < 0.01$, and ### $p < 0.001$ compared with the colitis group.
